# ULtiMATE System for Rapid Assembly of Customized TAL Effectors

**DOI:** 10.1371/journal.pone.0075649

**Published:** 2013-09-27

**Authors:** Junjiao Yang, Pengfei Yuan, Dingqiao Wen, Ying Sheng, Shiyou Zhu, Yuezhou Yu, Xiang Gao, Wensheng Wei

**Affiliations:** 1 State Key Laboratory of Protein and Plant Gene Research, College of Life Sciences, Peking University, Beijing, China; 2 Department of Electronics, School of Electronics Engineering and Computer Science, Peking University, Beijing, China; IGBMC/ICS, France

## Abstract

Engineered TAL-effector nucleases (TALENs) and TALE-based constructs have become powerful tools for eukaryotic genome editing. Although many methods have been reported, it remains a challenge for the assembly of designer-based TALE repeats in a fast, precise and cost-effective manner. We present an ULtiMATE (USER-based Ligation Mediated Assembly of TAL Effector) system for speedy and accurate assembly of customized TALE constructs. This method takes advantage of uracil-specific excision reagent (USER) to create multiple distinct sticky ends between any neighboring DNA fragments for specific ligation. With pre-assembled templates, multiple TALE DNA-binding domains could be efficiently assembled in order within hours with minimal manual operation. This system has been demonstrated to produce both functional TALENs for effective gene knockout and TALE-mediated gene-specific transcription activation (TALE-TA). The feature of both ease-of-operation and high efficiency of ULtiMATE system makes it not only an ideal method for biologic labs, but also an approach well suited for large-scale assembly of TALENs and any other TALE-based constructions.

## Introduction

Natural TAL effectors (TALEs), originally identified from bacteria *Xanthomonas* , mimic eukaryotic transcription factors to reprogram host cells [1]. A typical TALE contains a central DNA-binding region of tandem repeats of 34 amino acids, with each repeat specifically targeting a nucleotide using repeat variable diresidues (RVDs) at positions 12 and 13 [1,2]. This modular DNA-binding feature allows protein engineering by design-based assembly of TALE repeats for use in gene targeting [3,4]. The TAL effector-targeting domain has been shown to create site-specific DNA double-strand breaks (DSBs) when fused with the catalytic domain of the FokI nuclease [3,5-7]. Such TAL effector nucleases (TALENs) work in pairs each of which is designed to fuse the FokI monomer and binds opposing DNA target sites separated by an appropriative spacer. As a result, the two FokI monomers form a functional dimer to create a DSB. The cell’s DNA repair machinery activated by this DSB could give rise to nonhomologous end joining (NHEJ), leading to small insertions or deletions (indels) at or near the break site, resulting in disruption of gene function [3]. Alternatively, the homologous recombination (HR) could occur in the presence of DNA template that is identical or similar to the DNA surrounding the break site. Customized TALEs for specific gene activation have been achieved by fusion with the VP16 activation domain or its tetrameric derivative VP64 [3,8]. Targeted transcriptional repression was also achieved by a custom TALE targeting domain fused to a transcriptional repression domain in plant [9], as well as in human cell lines [10,11].

Although methods have been developed by many groups [4,6,8,12-19], it is still an engineering challenge for the assembly of design-based TALEs in a fast, reliable and cost-effective manner. Here we report a new approach, called ULtiMATE (USER-based Ligation Mediated Assembly of TAL Effector) system that is different from all reported protocols, most of which use variable forms of the Golden Gate cloning method [4,8,12,15,16]. ULtiMATE utilizes USER fusion technique [20], resulting in substantial reduction of workload and time span. The DNA fragments applicable to USER fusion were exclusively obtained from PCR reactions using special uracil-containing primers, which were performed with polymerases that could incorporate a deoxyadenine opposite a dU, such as PfuTurbo Cx Hotstart DNA polymerase. The USER^TM^ enzyme mix, a mixture of glycosidase (UDG) and DNA glycosylase-lyase endo VIII, was used to remove the dU residues to generate 3’-protruding sticky ends in PCR products [20]. 

## Materials and Methods

### Reagents

We used the following enzymes and kits: PfuTurbo Cx Hotstart DNA Polymerase (Agilent Technologies), T4 DNA ligase and USER^TM^ enzyme (New England Biolabs), BsmBI (Esp3I) (Thermo Scientific), T7 endonuclease I (New England Biolabs), EasyPure Quick Gel Extraction Kit and Trans1-T1 competent cells (Transgen), Xtreme Gene HP (Roche), 100-bp and 1-kb DNA ladders (Transgen). pGL3-TALEN vector (for TALEN) and pLentiLox3.7-TALE (for TALE-TA) were constructed for TALE cloning. Sequences of these two backbones were based on AvrBs3 TAL effector (*Xanthomonas campestris* pv. *vesicatoria* ) with 256/63 amino acids in the N/C-terminal of TALE repeats. Codons were optimized for mammalian expression. Detailed plasmid sequences were listed in the Supporting Information (Data S1).

#### Design and construction of four types of basic TALE repeat monomer (1-mers)

We designed four types of basic TALE repeat unit that differ in their DNA sequences flanking the RVD-coding region, designated as W-, X-, Y-, and Z-type, respectively. Albeit different in DNA sequences, these four types of TALE monomers encode the same amino acid sequence except for their RVD-coding region. Based on this design, total of 12 TAL effector repeat units were commercially synthesized in such a way that every type harbors the coding sequence of one of three RVDs that recognize a particular DNA base (NI->A, HD->C, NN->G and NG ->T). For user-friendly purpose, we have assigned each type with different colors, i.e., W-type in green, X-type in red, Y-type in blue and Z-type in yellow (Table S1A & B).

### Pre-assembly of 64 TALE repeat trimers (3-mers)

We pre-assembled all possible combination of TALE repeat trimers from the 12 monomers (Table S1C). This plasmid archive is used as the sole template pool to construct TALE repeats with desired length and module composition. The primers used for the PCR amplification of the monomers are described as below, and these TALE triplets were blunt-end ligated into pEASY-blunt vector (Transgen, CB101), followed by sequencing verification.

### Design of uracil primers used for PCR amplification of TALE repeat modules

The primers used for PCR amplification of the monomers or trimers are special uracil-containing primers commercially synthesized (Life Technologies). The primers are based on the four types of sequences, regardless of the RVDs-coding sequences they carry. The primer designation complies with the following simple rules: the capital letter refers to the module type (for both Forward and Reverse primers), and the next small letter refers to the module type upstream (for Forward primer) or downstream (for Reverse primer) of the current module. For the first repeat unit directly ligated to the N-terminal backbone, all the forward primers of four types (F-W5, F-X5, F-Y5, and F-Z5) carry an identical BsmBI site that ensures the module ligation with the corresponding N-terminal backbone. Similarly, all the reverse primers of four types (R-W3, R-X3, R-Y3 and R-Z3) for the last half repeat amplification carry an identical BsmBI site that is responsible for the specific ligation with the corresponding C-terminal backbone. The detailed design, designation and sequence information are listed in Table S2. For user-friendly purpose, we have assigned different colors to each letter that represents the module type, i.e., W & w in green, X & x in red, Y & y in blue, and, Z & z in yellow.

### Transfection of TALE constructs into mammalian cells

10^6^ of HeLa or HEK293T cells were electroporated with a pair of TALEN plasmids and pcDNA6HApuro vector (serving for marker selection) at 2:2:1 ratio (1 µg : 1 µg : 0.5 µg) using Lonza Nucleofector^TM^ following the manufacturer’s protocol. After 3 days of selection at 30°C with puromycin (2 µg/ml), resistant clones were cultured separately and further analyzed by genome PCR amplification. 10^6^ of HEK293T cells stably expressing miniCMV- Firefly luciferase reporters were cotransfected with TALE-

(RVD)_7,8,9_ plasmids and pRL-TK (expressing Renilla luciferase as internal control) at 10:1 ratio (2 sg : 0.2 µg) using Xtreme Gene HP (Roche) following the manufacturer’s protocol.

### Verification of the effects of TALE-TAs and TALENs

Cells transfected with TALE-TAs were harvested after 48 hours. Cell lysis and luciferase activity measurement were performed according to Dual-Luciferase Reporter Assay System (Promega, E1960). HeLa and HEK293Tcells transfected with TALENs were cultured in DMEM medium with 10% fetal bovine serum containing 2 µg/ml puromycin at 30°C for 3 days. Resistant cells were collected and genomic DNA was extracted with Qiagen DNeasy blood & tissue kit. PCR on genomic DNA was performed to amplify the region containing the TALENs target sequence. Primers used for different genes were listed in Table S3. PCR products were sequenced to calculate the rate of knockout events.

## Results and Discussion

It is difficult to assemble the TALE repeats because of their highly repetitive feature [1,2]. By leveraging codon degeneracy, we designed four types (W-, X-, Y-, and Z-type, respectively) of basic TALE units that encode the same amino acid sequence, but differ in DNA sequences flanking the RVD-coding region. The coding sequence of each DNA-recognition RVDs (NI, NN, HD or NG) was matched with three out of these four types, making a total of 12 basic TALE monomers (Table S1A and 1B). USER fusion technique [20] was employed to generate distinct ends between two neighboring repeats, in which the primers used for the PCR amplification of the TALE modules are special uracil-containing primers commercially synthesized. These PCR reactions must be performed with polymerases that could incorporate a deoxyadenine opposite a dU, such as PfuTurbo Cx Hotstart DNA polymerase. The USER^TM^ enzyme was used to remove the dU residues to generate 3’-protruding sticky ends in PCR products. Based on this system, primers were designed and commercially synthesized in such a way that the forward and reverse primers form 16 compatible pairs, each of which provides complementary sticky ends, created by USER^TM^ digestion of PCR products. The length of these 16 pairs of different sticky ends utilized for ligation ranges from 7 to 11 nt, sufficiently long to provide efficient ligation, and, at the same time, sufficiently diverse to minimize non-specific end joining (Table S2A and 2B). In addition, 4 pairs of primers harboring type IIs BsmBI (Esp3I) cutting sites were used to ligate end repeats of TALE arrays to the N- and C-terminal regions of the expression backbone (Table S2). Because the four forward primers that connect the N-terminal backbone yield the same sticky ends (5'-GAAC) after BsmBI digestion, and so do the four reverse primers (5'-AGCA) (Table S2B), ULtiMATE utilizes one universal backbone, unlike some other protocols that use four different backbones in order to match the last half repeats of the TALE arrays [4,8,12,16,17]. To further speed up the process, we pre-assembled an archive of 64 TALE trimers targeting all combination of 3-bp DNA, based on the following simple rules: 1) each trimer is composed of three different types of TALE monomers; and 2) each type (W, X, Y or Z) is uniformly distributed in the archive (Table S1C).

To assemble a TALE array targeting any particular DNA sequence, we first select the templates from the archive of 64 trimers (Table S1C). The choice of primers is based on the module types (W, X, Y or Z) between two neighboring PCR fragments for subsequent ligation (Table S2A). In particular, since we could, based on any trimer template, accurately acquire monomer, dimer or trimer through PCR by changing primers, we may produce TALE repeats with any number of monomers. The simple rule for the design is to conduct the least possible PCR reactions, while to ensure that all chosen primers for the same TALE assembly are unique. To facilitate the designing process, a JAVA program has been developed and attached (Supporting Information - Compressed/ZIP File Archive: Software S1.zip), which would output the design of all PCR reactions for a particular TALE assembly including the combination of templates and primers as well as the sequence of the final assembled TALE after the researcher simply inputs the TALE targeting sequence. The TALE cloning protocol for a single TALE array has been attached (Supporting Information – Methods S1).

**Figure 1:**
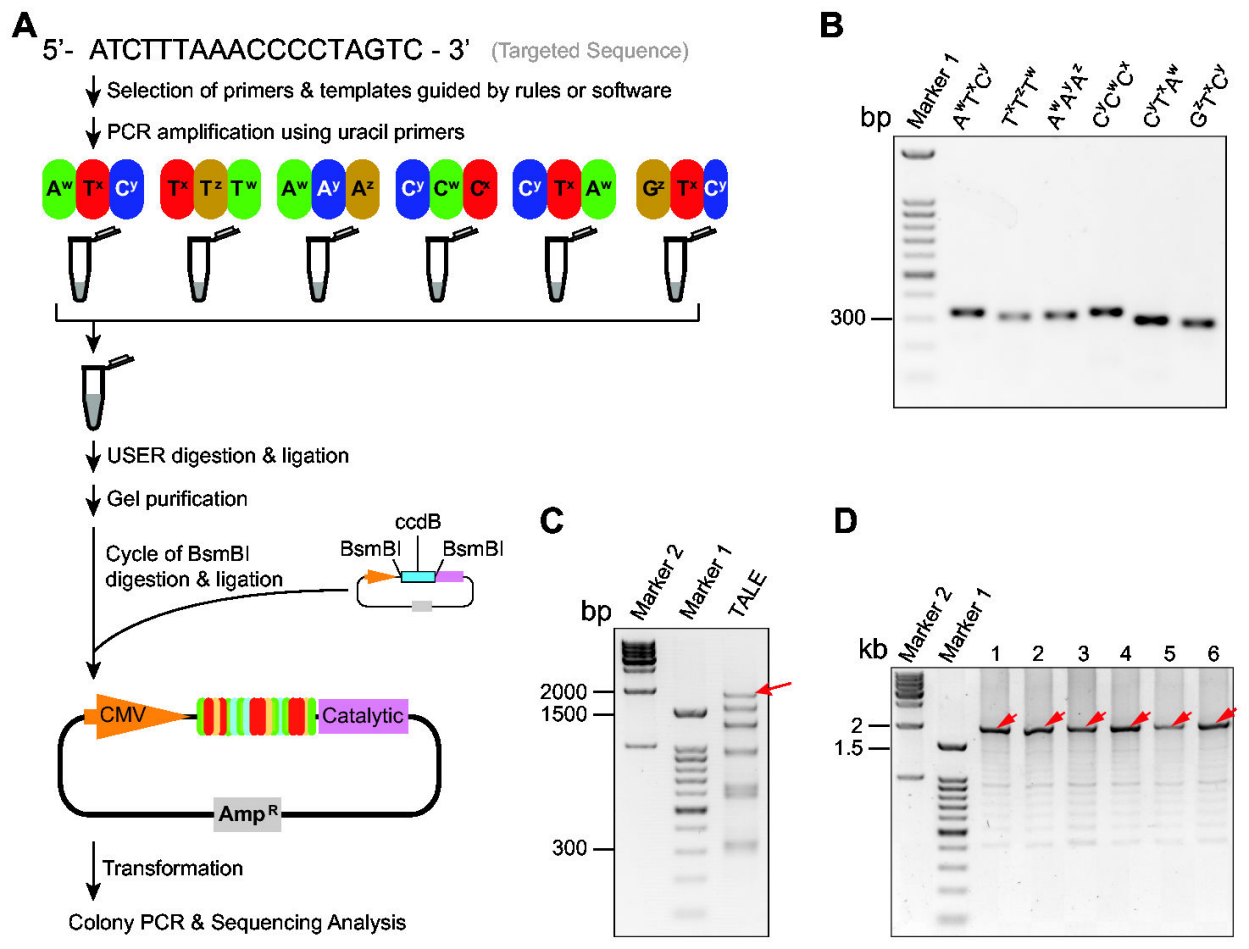
Customized TALE construction by ULtiMATE system. (**A**) Flowchart of the cloning process of a 17.5-mer TALE targeting ATCTTTAAACCCCTAGTC. The selection of templates and primers are based on the archive of 64 pre-assembled trimers and the 40-primer pool (Tables S1 and S2). The PCR amplification of trimers can be finished within 1.5 hrs. Without the need of purification, the PCR products of all reactions are mixed for subsequent USER^TM^ enzyme digestion and ligation sequentially in the same tube. The ligated fragments are gel-purified before mixed with TALE cloning backbone (Data S1B) for the cycle of BsmBI digestion and ligation, followed by the bacterial transformation (~ 1.5 hrs). The candidate clones are isolated based on colony PCR results, and verified by sequencing analysis. (**B**) PCR amplification of the trimers indicated by electrophoresis. Marker 1 is 100-bp DNA ladder (same below). (**C**) USER-mediated ligation indicated by electrophoresis. Red arrows refer to ligated DNA bands with correct sizes, ~1.8 kb. (**D**) Colony PCR of six randomly picked clones after transformation. Marker 2 is 1-kb DNA ladder. The DNA bands with correct size (~1.8 kb) are indicated by red arrows.

Figure 1 illustrated an example of TALE assembly (i.e. 17.5 repeats) by ULtiMATE system. Figure S1 showed examples of the design of PCR reactions using the JAVA program. After PCR reaction, the products were combined for USER^TM^ digestion and ligation, and the purification was unnecessary before or throughout the process. After the ligation, the assembled TALE arrays were gel purified and cloned through the Golden Gate method [16]. Because of the sequence discrepancy of the four types of monomers, PCR amplification of the 3-mers from the archive yielded a unique ~300-bp fragment (268-341 bp, depending on the primer pair), and the non-specific amplification of ~100-bp and ~200-bp fragments were rare (Figure 1B). The ligation efficiency and specificity of the USER-treated DNA fragments were high enough that these 6 end-compatible TALE triplets could be mixed for simultaneous ligation to produce 18-mer of TALE (Figure 1C), while the end-incompatible fragments could not under the same condition (data not shown). The success rate for colony PCR was 6/6 (Figure 1D) and 4/6 for sequencing verification. These two clones with sequencing errors all contained a single point mutation in the ligation region between two 3-mers, which were confirmed due to the synthetic error of primers. Indeed, this kind of error was completely eliminated when we used synthetic oligos with HPLC purification. From hundreds of TALE constructs we have assembled so far, the PCR errors were rare.

**Figure 2:**
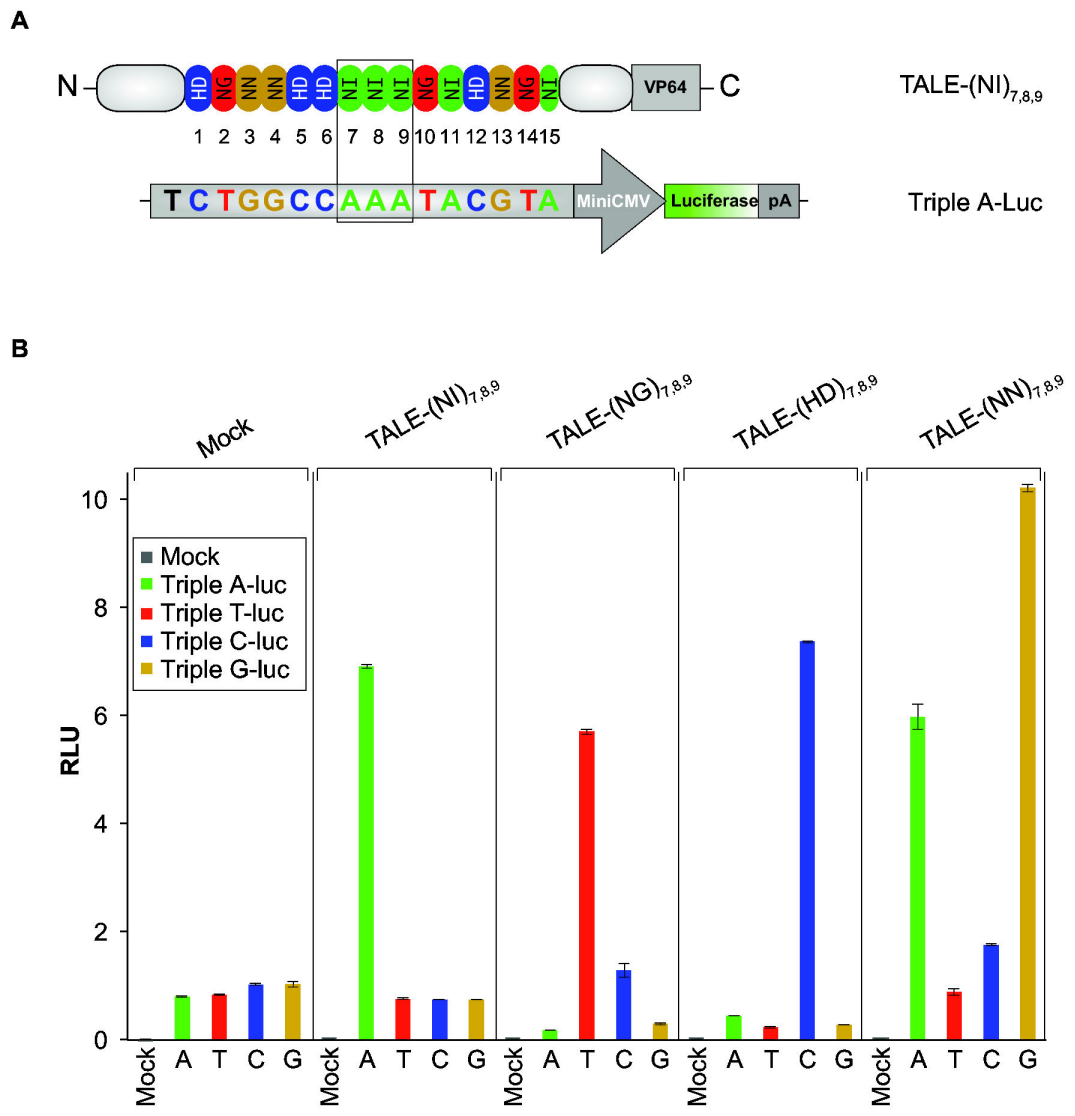
Effects of TALE-TAs in human cell lines. (**A**) Design and structure of a representative luciferase reporter, Triple A-Luc, and its corresponding TALE transactivator, TALE-(NI)_7,8,9_. The reporter contains an artificial TALE-binding sequence (CTGGCCAAATACGTA) upstream a mini CMV promoter, followed by the Firefly luciferase gene. TALE-(NI)_7,8,9_ carries the Triple A binding TALE, fused with VP64 viral activation domain. The TALE-binding sequences for Triple-A, -C, -G and -T reporters differ only in 3 consecutive bases in the middle. (**B**) The binding activity of each TALE-TAs is determined by measuring the relative luciferase units (RLU) for their corresponding reporter activity after normalization with a co-transfected Renilla expressing vector pRL-TK. Error bars indicate standard deviations of four replicates.

Concerned with the degenerate sequences used in the TALE units, we wanted to verify whether TALEs we produced have specific DNA binding capability. To test this, we constructed four luciferase reporters as well as their corresponding TALEs fused with VP64 (Figure 2). The reporters contained an artificial TALE-binding sequence upstream a mini CMV promoter, followed by firefly luciferase gene. The TALE-binding sequence for Triple-A reporter contains 3 consecutive A (i.e., CTGGCCAAATACGTA) from position 7 to 9 (Figure 2A). Likewise, Triple-C, -G and -T reporters differ only in these 3 consecutive bases. Co-introduced into cells, TALE-

(NI)_7,8,9_ could activate luciferase expression of Triple-A, but not the other reporters. Similarly, TALE-(HD)_7,8,9_ and TALE-(NG)_7,8,9_ only turned on C and T reporter, respectively, while TALE-(NN)_7,8,9_ could activate both A & G reporters (Figure 2B), thus perfectly matching the conventional RVDs-DNA recognition dogma. As all 12 basic TALE monomers were employed for above constructs, this result demonstrated that the TALE repeats made of ULtiMATE are fully functional in terms of the affinity and specificity of TALE-DNA recognition.

**Figure 3:**
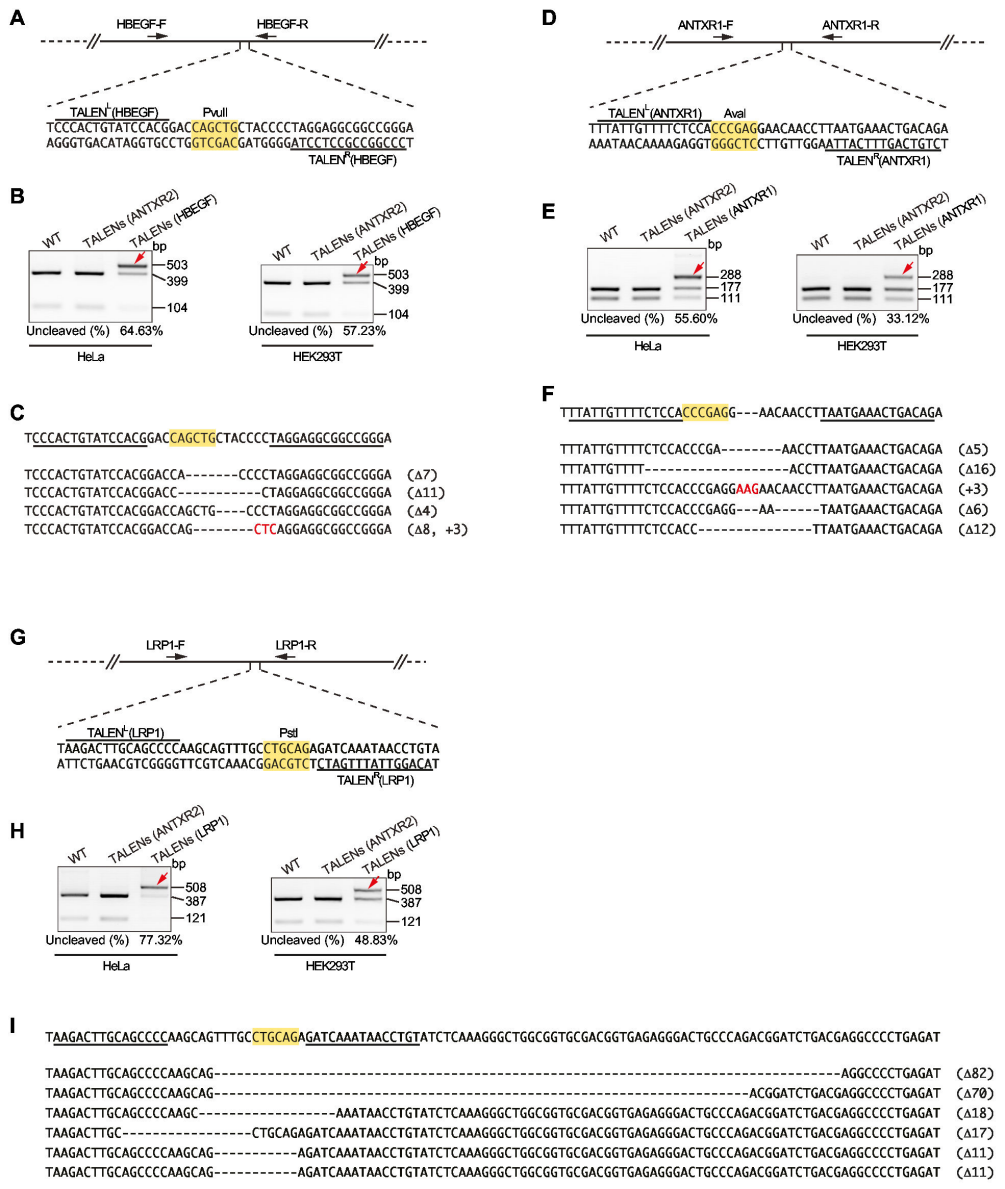
Examples of disruption of genes in human cell lines by ULtiMATE-engineered TALENs. (**A**, **D** and **G**) Partial sequences of *HBEGF*, *ANTXR1*, and *LRP1* genes in genome containing TALENs binding regions (overlined for TALEN^L^ and underlined for TALEN^R^). Restriction enzyme cutting sites are highlighted in yellow. (**B**, **E** and **H**) Measurement of indel rates in TALENs-treated HeLa and HEK293T cells by restriction enzyme digestion. The uncleaved bands indicate potential indels. Both wild type and cells treated by TALENs targeting *ANTXR2* gene are used as controls. The percentage of uncleaved band (indicated by red arrow) was measured using ImageJ (http://rsbweb.nih.gov/ij/). (**C**, **F** and **I**) Sequencing analysis of mutated alleles from 4-6 randomly selected TALENs clones (in HeLa cells). The TALENs binding sites (underlined) and restriction enzyme cutting sites (in yellow) are highlighted. Dashes and red letters indicate deletions and insertions, respectively.

We have made hundreds of TALENs constructs by ULtiMATE targeting a variety of human genes. Figure 3 illustrated the detailed analysis for gene knockout effect on three representative genes. The knockout efficiencies indicated by genome PCR and restriction enzyme digestion were 64.63% (57.23%), 55.60% (33.12%), and 77.32% (48.83%) in HeLa (HEK293T) cells for *HBEGF*, *ANTXR1*, and *LRP1*, respectively. Sequencing analysis of 4-7 randomly picked clones confirmed the frame shift-caused stop of gene expression by variable indels. Although the presence of a restriction site between two TALENs binding regions could help to confirm the occurrence of indels and determine the gene knockout efficiency, it is often problematic to include restriction enzyme cutting site(s) in TALEN targeting regions. Instead of conducting Surveyor Nuclease digestion [3], we found out that direct sequencing of the TALENs-targeting region of the pooled clones could also reveal such information if the indels frequency was over 15-20%. As shown in Figure S2, the appearance of the baseline noise in the four-color sequencing chromatogram indicated that the occurrence of indels, and the level of which correlated well with the efficiency of TALENs-mediated cleavage.

This ULtiMATE system has a number of unique advantages with reasonable cost (~ $15/clone in average, excluding labor and sequencing). The selection of primers and templates is self-explanatory, and the attached JAVA program would reduce the design effort to the minimal level. Without multi-rounds of enzyme digestion and purification, the whole process of ULtiMATE could be performed continuously in 1 test tube/well, which greatly simplifies the procedure and shortens the experimental time span. Our method makes the cloning of TALE comparable to a routine PCR-based cloning of any ~ 1.5- to 2.1-kb DNA fragment, only faster and easier. Although simple, ULtiMATE does not compromise accuracy and specificity, indicated by the success rate of colony PCR and sequencing verification. Overall, the high productivity of this protocol makes it realistic for average labs without liquid-handling robots to conduct medium to large scale synthesis of TALE repeats for massive amount of gene modification.

Because the USER-produced specific overhangs range from 7 to 11 nt, much longer than the 4-nt created by type IIs restriction enzymes (i.e., BsmBI) for Golden Gate fashion ligation, the ULtiMATE protocol has better efficiency that the ligation of up to 7-8 fragments could be performed in one reaction. In addition, since we could obtain monomer, 2-mer or 3-mer from any given 3-mer template, we were able to build TAL effector with different number of repeat units. It’s also feasible to assemble extra long TALE construct (up to 21-mers tested, data not shown) using ULtiMATE system. In this case, the PCR products were divided into different groups (under 7-8 fragments in each group) for the ligation and the subsequent purification steps.

Although ULtiMATE could use TALE monomer as the template, it is much more efficient to use the archive of 64 pre-assembled 3-mers instead. Because one RVD change affects 37 out of 64 triplets, it would not take long to upgrade the whole archive if necessary, especially when better RVDs, such as those for guanine-recognition, are discovered in the future. The high-throughput assembly methods have recently been reported [17,19], however, both FLASH assembly and the ligation-independent cloning (LIC) technique rely on large amount of preassembled units, limiting their broader usage for average labs.

The technology of TALE-mediated gene targeting has received tremendous attention due to its great potential in eukaryotic genome editing. We believe our ULtiMATE system could further unleash the power of this technique, especially for those who have the need to conduct medium- to large-scale TALE construction for massive gene targeting.

## Supporting Information

Data S1 **Sequences and structures of vectors / constructs. (A) pGL3-TALEN. (B) pLentiLox3.7-TALE. (C) pcDNA6-3A-luciferase.**
(PDF)

Figure S1 **Example of PCR design for ULtiMATE system using the JAVA program.**
(PDF)

Figure S2 **TALENs’ effects on patterns of sequencing chromatogram of targeted regions.** Partial sequences of seven representative genes (*ATG5*, *HBEGF*, *HSP90AB1*, *LRP1*, *PLXNA2*, *VPS15*, and *VPS34*) in TALENs targeting regions (underlined are binding sequences for TALEN^L^ and TALEN^R^) are indicated by the four-color sequencing chromatogram. The baseline noises indicate the occurrence of indels. The percentage of NHEJ induced indels was assayed using the mismatch-sensitive T7E1 endonuclease (Text S1) and quantified by ImageJ (http://rsbweb.nih.gov/ij/).
(PDF)

Methods S1 TALE cloning protocol for a single TALE array. (DOCX)

Software S1 User guideline. 1. Please install the latest version of JDK on your system (Windows, Mac, Linux and so on). The JDK can be downloaded from http://www.oracle.com/technetwork/java/javase/downloads/index.html. 2. For Mac or Linux users, please ensure your system has Jar Laucher. (Most of Mac or Linux already has it installed, but please double check.) 3. For Windows users, please double-click the file "ULtiMATE_PCR_Design_for_Windows.bat" to run the program; for Mac or Linux users, please run "ULtiMATE_PCR_Design.jar" using the Jar Launcher (right click and choose "Jar Launcher" to open it). 4. For Mac users, use Control + C/V to copy/paste in this software, and use Command + C/V to copy/paste in Mac environment.
(ZIP)

Table S1 Template preparation for PCR amplification of customized TAL effector repeat units. (PDF)

Table S2 Uracil primers used for PCR amplification of customized TALE repeats. (PDF)

Table S3 Primers for colony PCR of TALE constructs and genome PCR verification of TALENs-mediated. gene targeting.
(PDF)

Text S1 (DOCX)
