# Supplementary material for: ULtiMATE System for Rapid Assembly of Customized TAL Effectors
Source: PLoS One. 2013 Sep 27;8(9):e75649. doi: 10.1371/journal.pone.0075649 (PMC3815405; doi:10.1371/journal.pone.0075649)
Supplement: Data S1 — Sequences and structures of vectors / constructs. (A) pGL3-TALEN. (B) pLentiLox3.7-TALE. (C) pcDNA6-3A-luciferase. (PDF) [file pone.0075649.s001.pdf]

Supporting Information, Data S1

Sequences and structures of vectors / constructs. (A) pGL3-TALEN. (B) pLentiLox3.7-TALE. (C) pcDNA6-3A-luciferase.

(A) pGL3-TALEN:

```
   1 GGTACCGAGC TCTTACGCGT GCTAGCCCGG GCTCGAGATC TGCGATCTAA
  51 GTAAGCTTCG ATCTAAGTAA GCTTCGATGT ACGGGCCAGA TATACGCGTT
 101 GACATTGATT ATTGACTAGT TATTAATAGT AATCAATTAC GGGGTCATTA
 151 GTTCATAGCC CATATATGGA GTTCCGCGTT ACATAACTTA CGGTAAATGG
 201 CCCGCCTGGC TGACCGCCCA ACGACCCCCG CCCATTGACG TCAATAATGA
 251 CGTATGTTCC CATAGTAACG CCAATAGGGA CTTTCCATTG ACGTCAATGG
 301 GTGGAGTATT TACGGTAAAC TGCCCACTTG GCAGTACATC AAGTGTATCA
 351 TATGCCAAGT ACGCCCCCTA TTGACGTCAA TGACGGTAAA TGGCCCGCCT
 401 GGCATTATGC CCAGTACATG ACCTTATGGG ACTTTCCTAC TTGGCAGTAC
 451 ATCTACGTAT TAGTCATCGC TATTACCATG GTGATGCGGT TTTGGCAGTA
 501 CATCAATGGG CGTGGATAGC GGTTTGACTC ACGGGGATTT CCAAGTCTCC
 551 ACCCCATTGA CGTCAATGGG AGTTTGTTTT GGCACCAAAA TCAACGGGAC
 601 TTTCCAAAAT GTCGTAACAA CTCCGCCCCA TTGACGCAAA TGGGCGGTAG
 651 GCGTGTACGG TGGGAGGTCT ATATAAGCAG AGCTCTCTGG CTAACTAGAG
 701 AACCCACTGC TTACTGGCTT ATCGAAATTA ATACGACTCA CTATAGGGAG
 751 ACCCAAGCTG GCTAGCGAAG TTCCTATTCT CTAGAAAGTA TAGGAACTTC
 801 ATGGGCAGCC CCAAGAAGAA GAGAAAGGTG GGCAGCAAGA GAGACAGAAC
 851 CGGTAGGACC AGGCTGCCAT CTCCCCCTGC CCCTTCCCCC GCATTTAGCG
 901 CTGGGAGCTT TAGCGACCTG CTTAGGCAGT TCGACCCCAG CTTGTTCAAC
 951 ACCAGCCTGT TTGACAGCCT GCCTCCCTTC GGAGCGCACC ACACCGAAGC
1001 CGCCACCGGC GAGTGGGACG AGGTGCAAAG CGGCCTGAGG GCAGCGGACG
1051 CTCCTCCGCC AACCATGAGG GTGGCAGTGA CAGCAGCTAG GCCCCCTCGG
1101 GCAAAACCTG CACCCAGGAG AAGGGCTGCC CAACCCAGCG ACGCGAGTCC
1151 AGCCGCACAG GTGGACCTCA GGACGCTGGG CTACAGCCAG CAACAGCAAG
1201 AGAAGATCAA GCCCAAAGTA AGGAGCACCG TGGCCCAGCA CCACGAGGCC
1251 CTGGTGGGTC ACGGCTTCAC CCACGCGCAT ATCGTTGCTC TGAGCCAACA
1301 TCCCGCAGCT CTGGGTACCG TTGCGGTGAA GTATCAGGAC ATGATCGCGG
1351 CACTGCCTGA AGCTACACAC GAAGCCATAG TGGGCGTTGG CAAGCAGTGG
1401 AGCGGTGCCA GAGCGCTTGA GGCACTGTTG ACGGTGGCTG GCGAGCTGAG
1451 GGGACCGCCA CTGCAACTGG ACACCGGCCA ACTGCTGAAG ATCGCCAAGA
```

1501 GGGGAGGCGT GACGGCGGTG GAGGCCGTGC ATGCCTGGAG GAACGCACTG

1551 ACCGGCGCGC CCCTGAACAG AGACGCTGGC TTATCGAAAT TAATACGACT

1601 CACTATAGGG AGACCCAAGC TGGCTAGTTA AGCTATCAAC AAGTTTGTAC

1651 AAAAAAGCTG AACGAGAAAC GTAAAATGAT ATAAATATCA ATATATTAAA

1701 TTAGATTTTG CATAAAAAAC AGACTACATA ATACTGTAAA ACACAACATA

1751 TCCAGTCACT ATGAATCAAC TACTTAGATG GTATTAGTGA CCTGTAGTCG

1801 ACCGACAGCC TTCCAAATGT TCTTCGGGTG ATGCTGCCAA CTTAGTCGAC

1851 CGACAGCCTT CCAAATGTTC TTCTCAAACG GAATCGTCGT ATCCAGCCTA

1901 CTCGCTATTG TCCTCAATGC CGTATTAAAT CATAAAAAGA AATAAGAAAA

1951 AGAGGTGCGA GCCTCTTTTT TGTGTGACAA AATAAAAACA TCTACCTATT

2001 CATATACGCT AGTGTCATAG TCCTGAAAAT CATCTGCATC AAGAACAATT

2051 TCACAACTCT TATACTTTTC TCTTACAAGT CGTTCGGCTT CATCTGGATT

2101 TTCAGCCTCT ATACTTACTA AACGTGATAA AGTTTCTGTA ATTTCTACTG

2151 TATCGACCTG CAGACTGGCT GTGTATAAGG GAGCCTGACA TTTATATTCC

2201 CCAGAACATC AGGTTAATGG CGTTTTTGAT GTCATTTTCG CGGTGGCTGA

2251 GATCAGCCAC TTCTTCCCCG ATAACGGAGA CCGGCACACT GGCCATATCG

2301 GTGGTCATCA TGCGCCAGCT TTCATCCCCG ATATGCACCA CCGGGTAAAG

2351 TTCACGGGAG ACTTTATCTG ACAGCAGACG TGCACTGGCC AGGGGGATCA

2401 CCATCCGTCG CCCGGGCGTG TCAATAATAT CACTCTGTAC ATCCACAAAC

2451 AGACGATAAC GGCTCTCTCT TTTATAGGTG TAAACCTTAA ACTGCATTTC

2501 ACCAGCCCCT GTTCTCGTCA GCAAAAGAGC CGTTCATTTC AATAAACCGG

2551 GCGACCTCAG CCATCCCTTC CTGATTTTCC GCTTTCCAGC GTTCGGCACG

2601 CAGACGACGG GCTTCATTCT GCATGGTTGT GCTTACCAGA CCGGAGATAT

2651 TGACATCATA TATGCCTTGA GCAACTGATA GCTGTCGCTG TCAACTGTCA

2701 CTGTAATACG CTGCTTCATA GCATACCTCT TTTTGACATA CTTCGGGTAT

2751 ACATATCAGT ATATATTCTT ATACCGCAAA AATCAGCGCG CAAATACGCA

2801 TACTGTTATC TGGCTTTTAG TAAGCCGGAT CCACGCGGCG TTTACGCCCC

2851 CCCTGCCACT CATCGCAGTA CTGTTGTAAT TCATTAAGCA TTCTGCCGAC

2901 ATGGAAGCCA TCACAAACGG CATGATGAAC CTGAATCGCC AGCGGCATCA

2951 GCACCTTGTC GCCTTGCGTA TAATATTTGC CCATGGTGAA AACGGGGGCG

3001 AAGAAGTTGT CCATATTGGC CACGTTTAAA TCAAAACTGG TGAAACTCAC

3051 CCAGGGATTG GCTGAGACGA AAAACATATT CTCAATAAAC CCTTTAGGGA

3101 AATAGGCCAG GTTTTCACCG TAACACGCCA CATCTTGCGA ATATATGTGT

3151 AGAAACTGCC GGAAATCGTC GTGGTATTCA CTCCAGAGCG ATGAAAACGT

3201 TTCAGTTTGC TCATGGAAAA CGGTGTAACA AGGGTGAACA CTATCCCATA

3251 TCACCAGCTC ACCGTCTTTC ATTGCCATAC GGAATTCCGG ATGAGCATTC

3301 ATCAGGCGGG CAAGAATGTG AATAAAGGCC GGATAAAACT TGTGCTTATT

3351 TTTCTTTACG GTCTTTAAAA AGGCCGTAAT ATCCAGCTGA ACGGTCTGGT

3401 TATAGGTACA TTGAGCAACT GACTGAAATG CCTCAAAATG TTCTTTACGA

3451 TGCCATTGGG ATATATCAAC GGTGGTATAT CCAGTGATTT TTTTCTCCAT

3501 TTTAGCTTCC TTAGCTCCTG AAAATCTCGA TAACTCAAAA AATACGCCCG

3551 GTAGTGATCT TATTTCATTA TGGTGAAAGT TGGAACCTCT TACGTGCCGA

3601 TCAACGTCTC ATTTTCGCCA AAAGTTGGCC CAGGGCTTCC CGGTATCAAC

3651 AGGGACACCA GGATTTATTT ATTCTGCGAA GTGATCTTCC GTCACAGGTA

3701 TTTATTCGGC GCAAAGTGCG TCGGGTGATG CTGCCAACTT AGTCGACTAC

3751 AGGTCACTAA TACCATCTAA GTAGTTGATT CATAGTGACT GGATATGTTG

3801 TGTTTTACAG TATTATGTAG TCTGTTTTTT ATGCAAAATC TAATTTAATA

3851 TATTGATATT TATATCATTT TACGTTTCTC GTTCAGCTTT CTTGTACAAA

3901 GTGGTTGATC TAGAGGGCCC GCGGTTCGAA CGTCTCTAGC ATCGTGGCCC

3951 AGCTGAGCAG ACCCGACCCT GCCCTCGCCG CTCTGACCAA CGACCACCTG

4001 GTGGCCCTGG CTTGCCTCGG GGGCAGGCCA GCTCTTGACG CCGTGAAGAA

4051 GGGCCTTCCT CACGCCCCAG CCCTGATCAA GAGGACGAAC AGGAGGATCC

4101 CTGAGAGGAC CTCCCATAGG GTCGCCTTCA AGCAGCTAGT GAAATCTGAA

4151 TTGGAAGAGA AGAAATCTGA ACTTAGACAT AAATTGAAAT ATGTGCCACA

4201 TGAATATATT GAATTGATTG AAATCGCAAG AAATTCAACT CAGGATAGAA

4251 TCCTTGAAAT GAAGGTGATG GAGTTCTTTA TGAAGGTTTA TGGTTATCGT

4301 GGTAAACATT TGGGTGGATC AAGGAAACCA GACGGAGCAA TTTATACTGT

4351 CGGATCTCCT ATTGATTACG GTGTGATCGT TGATACTAAG GCATATTCAG

4401 GAGGTTATAA TCTTCCAATT GGTCAAGCAG ATGAAATGCA AAGATATGTC

4451 GAAGAGAATC AAACAAGAAA CAAGCATATC AACCCTAATG AATGGTGGAA

4501 AGTCTATCCA TCTTCAGTAA CAGAATTTAA GTTCTTGTTT GTGAGTGGTC

4551 ATTTCAAAGG AAACTACAAA GCTCAGCTTA CAAGATTGAA TCATATCACT

4601 AATTGTAATG GAGCTGTTCT TAGTGTAGAA GAGCTTTTGA TTGGTGGAGA

4651 AATGATTAAA GCTGGTACAT TGACACTTGA GGAAGTGAGA AGGAAATTTA

4701 ATAACGGTGA GATAAACTTT TAAAGATCTT AATCTAGAGT CGGGGCGGCC

4751 GGCCGCTTCG AGCAGACATG ATAAGATACA TTGATGAGTT TGGACAAACC

4801 ACAACTAGAA TGCAGTGAAA AAAATGCTTT ATTTGTGAAA TTTGTGATGC

4851 TATTGCTTTA TTTGTAACCA TTATAAGCTG CAATAAACAA GTTAACAACA

4901 ACAATTGCAT TCATTTTATG TTTCAGGTTC AGGGGGAGGT GTGGGAGGTT

4951 TTTTAAAGCA AGTAAAACCT CTACAAATGT GGTAAAATCG ATAAGGATCC

5001 GTCGACCGAT GCCCTTGAGA GCCTTCAACC CAGTCAGCTC CTTCCGGTGG

5051 GCGCGGGGCA TGACTATCGT CGCCGCACTT ATGACTGTCT TCTTTATCAT

5101 GCAACTCGTA GGACAGGTGC CGGCAGCGCT CTTCCGCTTC CTCGCTCACT

5151 GACTCGCTGC GCTCGGTCGT TCGGCTGCGG CGAGCGGTAT CAGCTCACTC

5201 AAAGGCGGTA ATACGGTTAT CCACAGAATC AGGGGATAAC GCAGGAAAGA

5251 ACATGTGAGC AAAAGGCCAG CAAAAGGCCA GGAACCGTAA AAAGGCCGCG

5301 TTGCTGGCGT TTTTCCATAG GCTCCGCCCC CCTGACGAGC ATCACAAAAA

5351 TCGACGCTCA AGTCAGAGGT GGCGAAACCC GACAGGACTA TAAAGATACC

5401 AGGCGTTTCC CCCTGGAAGC TCCCTCGTGC GCTCTCCTGT TCCGACCCTG

5451 CCGCTTACCG GATACCTGTC CGCCTTTCTC CCTTCGGGAA GCGTGGCGCT

5501 TTCTCATAGC TCACGCTGTA GGTATCTCAG TTCGGTGTAG GTCGTTCGCT

5551 CCAAGCTGGG CTGTGTGCAC GAACCCCCCG TTCAGCCCGA CCGCTGCGCC

5601 TTATCCGGTA ACTATCGTCT TGAGTCCAAC CCGGTAAGAC ACGACTTATC

5651 GCCACTGGCA GCAGCCACTG GTAACAGGAT TAGCAGAGCG AGGTATGTAG

5701 GCGGTGCTAC AGAGTTCTTG AAGTGGTGGC CTAACTACGG CTACACTAGA

5751 AGAACAGTAT TTGGTATCTG CGCTCTGCTG AAGCCAGTTA CCTTCGGAAA

5801 AAGAGTTGGT AGCTCTTGAT CCGGCAAACA AACCACCGCT GGTAGCGGTG

5851 GTTTTTTTGT TTGCAAGCAG CAGATTACGC GCAGAAAAAA AGGATCTCAA

5901 GAAGATCCTT TGATCTTTTC TACGGGGTCT GACGCTCAGT GGAACGAAAA

5951 CTCACGTTAA GGGATTTTGG TCATGAGATT ATCAAAAAGG ATCTTCACCT

6001 AGATCCTTTT AAATTAAAAA TGAAGTTTTA AATCAATCTA AAGTATATAT

6051 GAGTAAACTT GGTCTGACAG TTACCAATGC TTAATCAGTG AGGCACCTAT

6101 CTCAGCGATC TGTCTATTTC GTTCATCCAT AGTTGCCTGA CTCCCCGTCG

6151 TGTAGATAAC TACGATACGG GAGGGCTTAC CATCTGGCCC CAGTGCTGCA

6201 ATGATACCGC GAGACCCACG CTCACCGGCT CCAGATTTAT CAGCAATAAA

6251 CCAGCCAGCC GGAAGGGCCG AGCGCAGAAG TGGTCCTGCA ACTTTATCCG

6301 CCTCCATCCA GTCTATTAAT TGTTGCCGGG AAGCTAGAGT AAGTAGTTCG

6351 CCAGTTAATA GTTTGCGCAA CGTTGTTGCC ATTGCTACAG GCATCGTGGT

6401 GTCACGCTCG TCGTTTGGTA TGGCTTCATT CAGCTCCGGT TCCCAACGAT

6451 CAAGGCGAGT TACATGATCC CCCATGTTGT GCAAAAAAGC GGTTAGCTCC

6501 TTCGGTCCTC CGATCGTTGT CAGAAGTAAG TTGGCCGCAG TGTTATCACT

6551 CATGGTTATG GCAGCACTGC ATAATTCTCT TACTGTCATG CCATCCGTAA

6601 GATGCTTTTC TGTGACTGGT GAGTACTCAA CCAAGTCATT CTGAGAATAG

6651 TGTATGCGGC GACCGAGTTG CTCTTGCCCG GCGTCAATAC GGGATAATAC

6701 CGCGCCACAT AGCAGAACTT TAAAAGTGCT CATCATTGGA AAACGTTCTT

6751 CGGGGCGAAA ACTCTCAAGG ATCTTACCGC TGTTGAGATC CAGTTCGATG

6801 TAACCCACTC GTGCACCCAA CTGATCTTCA GCATCTTTTA CTTTCACCAG

6851 CGTTTCTGGG TGAGCAAAAA CAGGAAGGCA AAATGCCGCA AAAAAGGGAA

6901 TAAGGGCGAC ACGGAAATGT TGAATACTCA TACTCTTCCT TTTTCAATAT

6951 TATTGAAGCA TTTATCAGGG TTATTGTCTC ATGAGCGGAT ACATATTTGA

7001 ATGTATTTAG AAAAATAAAC AAATAGGGGT TCCGCGCACA TTTCCCCGAA

7051 AAGTGCCACC TGACGCGCCC TGTAGCGGCG CATTAAGCGC GGCGGGTGTG

7101 GTGGTTACGC GCAGCGTGAC CGCTACACTT GCCAGCGCCC TAGCGCCCGC

7151 TCCTTTCGCT TTCTTCCCTT CCTTTCTCGC CACGTTCGCC GGCTTTCCCC

7201 GTCAAGCTCT AAATCGGGGG CTCCCTTTAG GGTTCCGATT TAGTGCTTTA

7251 CGGCACCTCG ACCCCAAAAA ACTTGATTAG GGTGATGGTT CACGTAGTGG

7301 GCCATCGCCC TGATAGACGG TTTTTCGCCC TTTGACGTTG GAGTCCACGT

7351 TCTTTAATAG TGGACTCTTG TTCCAAACTG GAACAACACT CAACCCTATC

7401 TCGGTCTATT CTTTTGATTT ATAAGGGATT TTGCCGATTT CGGCCTATTG

7451 GTTAAAAAAT GAGCTGATTT AACAAAAATT TAACGCGAAT TTTAACAAAA

7501 TATTAACGCT TACAATTTGC CATTCGCCAT TCAGGCTGCG CAACTGTTGG

7551 GAAGGGCGAT CGGTGCGGGC CTCTTCGCTA TTACGCCAGC CCAAGCTACC

7601 ATGATAAGTA AGTAATATTA AGGTACGGGA GGTACTTGGA GCGGCCGCAA

7651 TAAAATATCT TTATTTTCAT TACATCTGTG TGTTGGTTTT TTGTGTGAAT

7701 CGATAGTACT AACATACGCT CTCCATCAAA ACAAAACGAA ACAAAACAAA

7751 CTAGCAAAAT AGGCTGTCCC CAGTGCAAGT GCAGGTGCCA GAACATTTCT

7801 CTATCGATA

Features:

75 – 729 bp CMV promoter

804 – 854 bp NLS

801 – 1568 bp TALE N-terminal

1565 – 1575 bp BsmBI

1576 – 3937 bp CmR-ccdB

3932 – 3941 bp BsmBI

3938 – 4126 bp TALE C-terminal

4127 – 4732 bp FokI

(B) pLentiLox3.7-TALE:

1 GTCGACGGAT CGGGAGATCT CCCGATCCCC TATGGTGCAC TCTCAGTACA

51 ATCTGCTCTG ATGCCGCATA GTTAAGCCAG TATCTGCTCC CTGCTTGTGT

101 GTTGGAGGTC GCTGAGTAGT GCGCGAGCAA AATTTAAGCT ACAACAAGGC

151 AAGGCTTGAC CGACAATTGC ATGAAGAATC TGCTTAGGGT TAGGCGTTTT

201 GCGCTGCTTC GCGATGTACG GGCCAGATAT ACGCGTTGAC ATTGATTATT

251 GACTAGTTAT TAATAGTAAT CAATTACGGG GTCATTAGTT CATAGCCCAT

301 ATATGGAGTT CCGCGTTACA TAACTTACGG TAAATGGCCC GCCTGGCTGA

351 CCGCCCAACG ACCCCCGCCC ATTGACGTCA ATAATGACGT ATGTTCCCAT

401 AGTAACGCCA ATAGGGACTT TCCATTGACG TCAATGGGTG GAGTATTTAC

451 GGTAAACTGC CCACTTGGCA GTACATCAAG TGTATCATAT GCCAAGTACG

501 CCCCCTATTG ACGTCAATGA CGGTAAATGG CCCGCCTGGC ATTATGCCCA

551 GTACATGACC TTATGGGACT TTCCTACTTG GCAGTACATC TACGTATTAG

601 TCATCGCTAT TACCATGGTG ATGCGGTTTT GGCAGTACAT CAATGGGCGT

651 GGATAGCGGT TTGACTCACG GGGATTTCCA AGTCTCCACC CCATTGACGT

701 CAATGGGAGT TTGTTTTGGC ACCAAAATCA ACGGGACTTT CCAAAATGTC

751 GTAACAACTC CGCCCCATTG ACGCAAATGG GCGGTAGGCG TGTACGGTGG

801 GAGGTCTATA TAAGCAGCGC GTTTTGCCTG TACTGGGTCT CTCTGGTTAG

851 ACCAGATCTG AGCCTGGGAG CTCTCTGGCT AACTAGGGAA CCCACTGCTT

901 AAGCCTCAAT AAAGCTTGCC TTGAGTGCTT CAAGTAGTGT GTGCCCGTCT

951 GTTGTGTGAC TCTGGTAACT AGAGATCCCT CAGACCCTTT TAGTCAGTGT

1001 GGAAAATCTC TAGCAGTGGC GCCCGAACAG GGACTTGAAA GCGAAAGGGA

1051 AACCAGAGGA GCTCTCTCGA CGCAGGACTC GGCTTGCTGA AGCGCGCACG

1101 GCAAGAGGCG AGGGGCGGCG ACTGGTGAGT ACGCCAAAAA TTTTGACTAG

1151 CGGAGGCTAG AAGGAGAGAG ATGGGTGCGA GAGCGTCAGT ATTAAGCGGG

1201 GGAGAATTAG ATCGCGATGG GAAAAAATTC GGTTAAGGCC AGGGGGAAAG

1251 AAAAAATATA AATTAAAACA TATAGTATGG GCAAGCAGGG AGCTAGAACG

1301 ATTCGCAGTT AATCCTGGCC TGTTAGAAAC ATCAGAAGGC TGTAGACAAA

1351 TACTGGGACA GCTACAACCA TCCCTTCAGA CAGGATCAGA AGAACTTAGA

1401 TCATTATATA ATACAGTAGC AACCCTCTAT TGTGTGCATC AAAGGATAGA

1451 GATAAAAGAC ACCAAGGAAG CTTTAGACAA GATAGAGGAA GAGCAAAACA

1501 AAAGTAAGAC CACCGCACAG CAAGCGGCCG GCCGCGCTGA TCTTCAGACC

1551 TGGAGGAGGA GATATGAGGG ACAATTGGAG AAGTGAATTA TATAAATATA

1601 AAGTAGTAAA AATTGAACCA TTAGGAGTAG CACCCACCAA GGCAAAGAGA

1651 AGAGTGGTGC AGAGAGAAAA AAGAGCAGTG GGAATAGGAG CTTTGTTCCT

1701 TGGGTTCTTG GGAGCAGCAG GAAGCACTAT GGGCGCAGCG TCAATGACGC

1751 TGACGGTACA GGCCAGACAA TTATTGTCTG GTATAGTGCA GCAGCAGAAC

1801 AATTTGCTGA GGGCTATTGA GGCGCAACAG CATCTGTTGC AACTCACAGT

1851 CTGGGGCATC AAGCAGCTCC AGGCAAGAAT CCTGGCTGTG GAAAGATACC

1901 TAAAGGATCA ACAGCTCCTG GGGATTTGGG GTTGCTCTGG AAAACTCATT

1951 TGCACCACTG CTGTGCCTTG GAATGCTAGT TGGAGTAATA AATCTCTGGA

2001 ACAGATTTGG AATCACACGA CCTGGATGGA GTGGGACAGA GAAATTAACA

2051 ATTACACAAG CTTAATACAC TCCTTAATTG AAGAATCGCA AAACCAGCAA

2101 GAAAAGAATG AACAAGAATT ATTGGAATTA GATAAATGGG CAAGTTTGTG

2151 GAATTGGTTT AACATAACAA ATTGGCTGTG GTATATAAAA TTATTCATAA

2201 TGATAGTAGG AGGCTTGGTA GGTTTAAGAA TAGTTTTTGC TGTACTTTCT

2251 ATAGTGAATA GAGTTAGGCA GGGATATTCA CCATTATCGT TTCAGACCCA

2301 CCTCCCAACC CCGAGGGGAC CCGACAGGCC CGAAGGAATA GAAGAAGAAG

2351 GTGGAGAGAG AGACAGAGAC AGATCCATTC GATTAGTGAA CGGATCGGCA

2401 CTGCGTGCGC CAATTCTGCA GACAAATGGC AGTATTCATC CACAATTTTA

2451 AAAGAAAAGG GGGGATTGGG GGGTACAGTG CAGGGGAAAG AATAGTAGAC

2501 ATAATAGCAA CAGACATACA AACTAAAGAA TTACAAAAAC AAATTACAAA

2551 AATTCAAAAT TTTCGGGTTT ATTACAGGGA CAGCAGAGAT CCAGTTTGGT

2601 TAGTACCGGG CCCGCTCTAG ACGATGTACG GGCCAGATAT ACGCGTTGAC

2651 ATTGATTATT GACTAGTTAT TAATAGTAAT CAATTACGGG GTCATTAGTT

2701 CATAGCCCAT ATATGGAGTT CCGCGTTACA TAACTTACGG TAAATGGCCC

2751 GCCTGGCTGA CCGCCCAACG ACCCCCGCCC ATTGACGTCA ATAATGACGT

2801 ATGTTCCCAT AGTAACGCCA ATAGGGACTT TCCATTGACG TCAATGGGTG

2851 GAGTATTTAC GGTAAACTGC CCACTTGGCA GTACATCAAG TGTATCATAT

2901 GCCAAGTACG CCCCCTATTG ACGTCAATGA CGGTAAATGG CCCGCCTGGC

2951 ATTATGCCCA GTACATGACC TTATGGGACT TTCCTACTTG GCAGTACATC

3001 TACGTATTAG TCATCGCTAT TACCATGGTG ATGCGGTTTT GGCAGTACAT

3051 CAATGGGCGT GGATAGCGGT TTGACTCACG GGGATTTCCA AGTCTCCACC

3101 CCATTGACGT CAATGGGAGT TTGTTTTGGC ACCAAAATCA ACGGGACTTT

3151 CCAAAATGTC GTAACAACTC CGCCCCATTG ACGCAAATGG GCGGTAGGCG

3201 TGTACGGTGG GAGGTCTATA TAAGCAGAGC TCTCTGGCTA ACTAGAGAAC

3251 CCACTGCTTA CTGGCTTATC GAAATTAATA CGACTCACTA TAGGGAGACC

3301 CAAGCTGGCT AGCGAAGTTC CTATTCTCTA GAAAGTATAG GAACTTCATG

3351 AGGACCAGGC TGCCATCTCC CCCTGCCCCT TCCCCCGCAT TTAGCGCTGG

3401 GAGCTTTAGC GACCTGCTTA GGCAGTTCGA CCCCAGCTTG TTCAACACCA

3451 GCCTGTTTGA CAGCCTGCCT CCCTTCGGAG CGCACCACAC CGAAGCCGCC

3501 ACCGGCGAGT GGGACGAGGT GCAAAGCGGC CTGAGGGCAG CGGACGCTCC

3551 TCCGCCAACC ATGAGGGTGG CAGTGACAGC AGCTAGGCCC CCTCGGGCAA

3601 AACCTGCACC CAGGAGAAGG GCTGCCCAAC CCAGCGACGC GAGTCCAGCC

3651 GCACAGGTGG ACCTCAGGAC GCTGGGCTAC AGCCAGCAAC AGCAAGAGAA

3701 GATCAAGCCC AAAGTAAGGA GCACCGTGGC CCAGCACCAC GAGGCCCTGG

3751 TGGGTCACGG CTTCACCCAC GCGCATATCG TTGCTCTGAG CCAACATCCC

3801 GCAGCTCTGG GTACCGTTGC GGTGAAGTAT CAGGACATGA TCGCGGCACT

3851 GCCTGAAGCT ACACACGAAG CCATAGTGGG CGTTGGCAAG CAGTGGAGCG

3901 GTGCCAGAGC GCTTGAGGCA CTGTTGACGG TGGCTGGCGA GCTGAGGGGA

3951 CCGCCACTGC AACTGGACAC CGGCCAACTG CTGAAGATCG CCAAGAGGGG

4001 AGGCGTGACG GCGGTGGAGG CCGTGCATGC CTGGAGGAAT GCCCTGACCG

4051 GCGCGCCCCT GAACAGAGAC GCTGGCTTAT CGAAATTAAT ACGACTCACT

4101 ATAGGGAGAC CCAAGCTGGC TAGTTAAGCT ATCAACAAGT TTGTACAAAA

4151 AAGCTGAACG AGAAACGTAA AATGATATAA ATATCAATAT ATTAAATTAG

4201 ATTTTGCATA AAAAACAGAC TACATAATAC TGTAAAACAC AACATATCCA

4251 GTCACTATGA ATCAACTACT TAGATGGTAT TAGTGACCTG TAGTCGACCG

4301 ACAGCCTTCC AAATGTTCTT CGGGTGATGC TGCCAACTTA GTCGACCGAC

4351 AGCCTTCCAA ATGTTCTTCT CAAACGGAAT CGTCGTATCC AGCCTACTCG

4401 CTATTGTCCT CAATGCCGTA TTAAATCATA AAAAGAAATA AGAAAAAGAG

4451 GTGCGAGCCT CTTTTTTGTG TGACAAAATA AAAACATCTA CCTATTCATA

4501 TACGCTAGTG TCATAGTCCT GAAAATCATC TGCATCAAGA ACAATTTCAC

4551 AACTCTTATA CTTTTCTCTT ACAAGTCGTT CGGCTTCATC TGGATTTTCA

4601 GCCTCTATAC TTACTAAACG TGATAAAGTT TCTGTAATTT CTACTGTATC

4651 GACCTGCAGA CTGGCTGTGT ATAAGGGAGC CTGACATTTA TATTCCCCAG

4701 AACATCAGGT TAATGGCGTT TTTGATGTCA TTTTCGCGGT GGCTGAGATC

4751 AGCCACTTCT TCCCCGATAA CGGAGACCGG CACACTGGCC ATATCGGTGG

4801 TCATCATGCG CCAGCTTTCA TCCCCGATAT GCACCACCGG GTAAAGTTCA

4851 CGGGAGACTT TATCTGACAG CAGACGTGCA CTGGCCAGGG GGATCACCAT

4901 CCGTCGCCCG GGCGTGTCAA TAATATCACT CTGTACATCC ACAAACAGAC

4951 GATAACGGCT CTCTCTTTTA TAGGTGTAAA CCTTAAACTG CATTTCACCA

5001 GCCCCTGTTC TCGTCAGCAA AAGAGCCGTT CATTTCAATA AACCGGGCGA

5051 CCTCAGCCAT CCCTTCCTGA TTTTCCGCTT TCCAGCGTTC GGCACGCAGA

5101 CGACGGGCTT CATTCTGCAT GGTTGTGCTT ACCAGACCGG AGATATTGAC

5151 ATCATATATG CCTTGAGCAA CTGATAGCTG TCGCTGTCAA CTGTCACTGT

5201 AATACGCTGC TTCATAGCAT ACCTCTTTTT GACATACTTC GGGTATACAT

5251 ATCAGTATAT ATTCTTATAC CGCAAAAATC AGCGCGCAAA TACGCATACT

5301 GTTATCTGGC TTTTAGTAAG CCGGATCCAC GCGGCGTTTA CGCCCCCCCT

5351 GCCACTCATC GCAGTACTGT TGTAATTCAT TAAGCATTCT GCCGACATGG

5401 AAGCCATCAC AAACGGCATG ATGAACCTGA ATCGCCAGCG GCATCAGCAC

5451 CTTGTCGCCT TGCGTATAAT ATTTGCCCAT GGTGAAAACG GGGGCGAAGA

5501 AGTTGTCCAT ATTGGCCACG TTTAAATCAA AACTGGTGAA ACTCACCCAG

5551 GGATTGGCTG AGACGAAAAA CATATTCTCA ATAAACCCTT TAGGGAAATA

5601 GGCCAGGTTT TCACCGTAAC ACGCCACATC TTGCGAATAT ATGTGTAGAA

5651 ACTGCCGGAA ATCGTCGTGG TATTCACTCC AGAGCGATGA AAACGTTTCA

5701 GTTTGCTCAT GGAAAACGGT GTAACAAGGG TGAACACTAT CCCATATCAC

5751 CAGCTCACCG TCTTTCATTG CCATACGGAA TTCCGGATGA GCATTCATCA

5801 GGCGGGCAAG AATGTGAATA AAGGCCGGAT AAAACTTGTG CTTATTTTTC

5851 TTTACGGTCT TTAAAAAGGC CGTAATATCC AGCTGAACGG TCTGGTTATA

5901 GGTACATTGA GCAACTGACT GAAATGCCTC AAAATGTTCT TTACGATGCC

5951 ATTGGGATAT ATCAACGGTG GTATATCCAG TGATTTTTTT CTCCATTTTA

6001 GCTTCCTTAG CTCCTGAAAA TCTCGATAAC TCAAAAAATA CGCCCGGTAG

6051 TGATCTTATT TCATTATGGT GAAAGTTGGA ACCTCTTACG TGCCGATCAA

6101 CGTCTCATTT TCGCCAAAAG TTGGCCCAGG GCTTCCCGGT ATCAACAGGG

6151 ACACCAGGAT TTATTTATTC TGCGAAGTGA TCTTCCGTCA CAGGTATTTA

6201 TTCGGCGCAA AGTGCGTCGG GTGATGCTGC CAACTTAGTC GACTACAGGT

6251 CACTAATACC ATCTAAGTAG TTGATTCATA GTGACTGGAT ATGTTGTGTT

6301 TTACAGTATT ATGTAGTCTG TTTTTTATGC AAAATCTAAT TTAATATATT

6351 GATATTTATA TCATTTTACG TTTCTCGTTC AGCTTTCTTG TACAAAGTGG

6401 TTGATCTAGA GGGCCCGCGG TTCGAACGTC TCTAGCATCG TGGCCCAGCT

6451 GTCTCGGCCC GACCCTGCCC TCGCCGCTCT GACCAACGAC CACCTGGTGG

6501 CCCTGGCTTG CCTCGGGGGC AGGCCAGCTC TTGACGCCGT GAAGAAGGGC

6551 CTTCCTCACG CCCCAGCCCT GATCAAGCGG ACCAACAGAA GGATTCCCGA

6601 GAGGACATCA CATCGAGTGG CAGATCACGC GCAAGTGGTC CGCGTGCTCG

6651 GATTCTTCCA GTGTCACTCC CACCCCGCAC AAGCGTTCGA TGACGCCATG

6701 ACTCAATTTG GTATGTCGAG ACACGGACTG CTGCAGCTCT TTCGTAGAGT

6751 CGGTGTCACA GAACTCGAGG CCCGCTCGGG CACACTGCCT CCCGCCTCCC

6801 AGCGGTGGGA CAGGATTCTC CAAGCGAGCG GTATGAAACG CGCGAAGCCT

6851 TCACCTACGT CAACTCAGAC ACCTGACCAG GCGAGCCTTC ATGCGTTCGC

6901 AGACTCGCTG GAGAGGGATT TGGACGCGCC CTCGCCCATG CATGAAGGGG

6951 ACCAAACTCG CGCGTCAGCT AGCCCCAAGA AGAAGAGAAA GGTGGAGGCC

7001 AGCGGTTCCG GACGGGCTGA CGCATTGGAC GATTTTGATC TGGATATGCT

7051 GGGAAGTGAC GCCCTCGATG ATTTTGACCT TGACATGCTT GGTTCGGATG

7101 CCCTTGATGA CTTTGACCTC GACATGCTCG GCAGTGACGC CCTTGATGAT

7151 TTCGACCTGG ACATGCTGAT TAACTCTAGA GGCAGTGGAG AGGGCAGAGG

7201 AAGTCTGCTA ACATGCGGTG ACGTCGAGGA GAATCCTGGC CCAGTGAGCA

7251 AGGGCGAGGA GGATAACATG GCCATCATCA AGGAGTTCAT GCGCTTCAAG

7301 GTGCACATGG AGGGCTCCGT GAACGGCCAC GAGTTCGAGA TCGAGGGCGA

7351 GGGCGAGGGC CGCCCCTACG AGGGCACCCA GACCGCCAAG CTGAAGGTGA

7401 CCAAGGGTGG CCCCCTGCCC TTCGCCTGGG ACATCCTGTC CCCTCAGTTC

7451 ATGTACGGCT CCAAGGCCTA CGTGAAGCAC CCCGCCGACA TCCCCGACTA

7501 CTTGAAGCTG TCCTTCCCCG AGGGCTTCAA GTGGGAGCGC GTGATGAACT

7551 TCGAGGACGG CGGCGTGGTG ACCGTGACCC AGGACTCCTC CCTGCAGGAC

7601 GGCGAGTTCA TCTACAAGGT GAAGCTGCGC GGCACCAACT TCCCCTCCGA

7651 CGGCCCCGTA ATGCAGAAGA AGACCATGGG CTGGGAGGCC TCCTCCGAGC

7701 GGATGTACCC CGAGGACGGC GCCCTGAAGG GCGAGATCAA GCAGAGGCTG

7751 AAGCTGAAGG ACGGCGGCCA CTACGACGCT GAGGTCAAGA CCACCTACAA

7801 GGCCAAGAAG CCCGTGCAGC TGCCCGGCGC CTACAACGTC AACATCAAGT

7851 TGGACATCAC CTCCCACAAC GAGGACTACA CCATCGTGGA ACAGTACGAA

7901 CGCGCCGAGG GCCGCCACTC CACCGGCGGC ATGGACGAGC TGTACAAGTA

7951 ACATGTTTAA GGGTTCCGGT TCCACTAGGT ACAATTCGAT ATCAAGCTTA

8001 TCGATAATCA ACCTCTGGAT TACAAAATTT GTGAAAGATT GACTGGTATT

8051 CTTAACTATG TTGCTCCTTT TACGCTATGT GGATACGCTG CTTTAATGCC

8101 TTTGTATCAT GCTATTGCTT CCCGTATGGC TTTCATTTTC TCCTCCTTGT

8151 ATAAATCCTG GTTGCTGTCT CTTTATGAGG AGTTGTGGCC CGTTGTCAGG

8201 CAACGTGGCG TGGTGTGCAC TGTGTTTGCT GACGCAACCC CCACTGGTTG

8251 GGGCATTGCC ACCACCTGTC AGCTCCTTTC CGGGACTTTC GCTTTCCCCC

8301 TCCCTATTGC CACGGCGGAA CTCATCGCCG CCTGCCTTGC CCGCTGCTGG

8351 ACAGGGGCTC GGCTGTTGGG CACTGACAAT TCCGTGGTGT TGTCGGGGAA

8401 ATCATCGTCC TTTCCTTGGC TGCTCGCCTG TGTTGCCACC TGGATTCTGC

8451 GCGGGACGTC CTTCTGCTAC GTCCCTTCGG CCCTCAATCC AGCGGACCTT

8501 CCTTCCCGCG GCCTGCTGCC GGCTCTGCGG CCTCTTCCGC GTCTTCGCCT

8551 TCGCCCTCAG ACGAGTCGGA TCTCCCTTTG GGCCGCCTCC CCGCATCGAT

8601 ACCGTCGACC TCGATCGAGA CCTAGAAAAA CATGGAGCAA TCACAAGTAG

8651 CAATACAGCA GCTACCAATG CTGATTGTGC CTGGCTAGAA GCACAAGAGG

8701 AGGAGGAGGT GGGTTTTCCA GTCACACCTC AGGTACCTTT AAGACCAATG

8751 ACTTACAAGG CAGCTGTAGA TCTTAGCCAC TTTTTAAAAG AAAAGGGGGG

8801 ACTGGAAGGG CTAATTCACT CCCAACGAAG ACAAGATATC CTTGATCTGT

8851 GGATCTACCA CACACAAGGC TACTTCCCTG ATTGGCAGAA CTACACACCA

8901 GGGCCAGGGA TCAGATATCC ACTGACCTTT GGATGGTGCT ACAAGCTAGT

8951 ACCAGTTGAG CAAGAGAAGG TAGAAGAAGC CAATGAAGGA GAGAACACCC

9001 GCTTGTTACA CCCTGTGAGC CTGCATGGGA TGGATGACCC GGAGAGAGAA

9051 GTATTAGAGT GGAGGTTTGA CAGCCGCCTA GCATTTCATC ACATGGCCCG

9101 AGAGCTGCAT CCGGACTGTA CTGGGTCTCT CTGGTTAGAC CAGATCTGAG

9151 CCTGGGAGCT CTCTGGCTAA CTAGGGAACC CACTGCTTAA GCCTCAATAA

9201 AGCTTGCCTT GAGTGCTTCA AGTAGTGTGT GCCCGTCTGT TGTGTGACTC

9251 TGGTAACTAG AGATCCCTCA GACCCTTTTA GTCAGTGTGG AAAATCTCTA

9301 GCAGCATGTG AGCAAAAGGC CAGCAAAAGG CCAGGAACCG TAAAAAGGCC

9351 GCGTTGCTGG CGTTTTTCCA TAGGCTCCGC CCCCCTGACG AGCATCACAA

9401 AAATCGACGC TCAAGTCAGA GGTGGCGAAA CCCGACAGGA CTATAAAGAT

9451 ACCAGGCGTT TCCCCCTGGA AGCTCCCTCG TGCGCTCTCC TGTTCCGACC

9501 CTGCCGCTTA CCGGATACCT GTCCGCCTTT CTCCCTTCGG GAAGCGTGGC

9551 GCTTTCTCAT AGCTCACGCT GTAGGTATCT CAGTTCGGTG TAGGTCGTTC

9601 GCTCCAAGCT GGGCTGTGTG CACGAACCCC CCGTTCAGCC CGACCGCTGC

9651 GCCTTATCCG GTAACTATCG TCTTGAGTCC AACCCGGTAA GACACGACTT

9701 ATCGCCACTG GCAGCAGCCA CTGGTAACAG GATTAGCAGA GCGAGGTATG

9751 TAGGCGGTGC TACAGAGTTC TTGAAGTGGT GGCCTAACTA CGGCTACACT

9801 AGAAGAACAG TATTTGGTAT CTGCGCTCTG CTGAAGCCAG TTACCTTCGG

9851 AAAAAGAGTT GGTAGCTCTT GATCCGGCAA ACAAACCACC GCTGGTAGCG

9901 GTGGTTTTTT TGTTTGCAAG CAGCAGATTA CGCGCAGAAA AAAAGGATCT

9951 CAAGAAGATC CTTTGATCTT TTCTACGGGG TCTGACGCTC AGTGGAACGA

10001 AAACTCACGT TAAGGGATTT TGGTCATGAG ATTATCAAAA AGGATCTTCA

10051 CCTAGATCCT TTTAAATTAA AAATGAAGTT TTAAATCAAT CTAAAGTATA

10101 TATGAGTAAA CTTGGTCTGA CAGTTACCAA TGCTTAATCA GTGAGGCACC

10151 TATCTCAGCG ATCTGTCTAT TTCGTTCATC CATAGTTGCC TGACTCCCCG

10201 TCGTGTAGAT AACTACGATA CGGGAGGGCT TACCATCTGG CCCCAGTGCT

10251 GCAATGATAC CGCGAGACCC ACGCTCACCG GCTCCAGATT TATCAGCAAT

10301 AAACCAGCCA GCCGGAAGGG CCGAGCGCAG AAGTGGTCCT GCAACTTTAT

10351 CCGCCTCCAT CCAGTCTATT AATTGTTGCC GGGAAGCTAG AGTAAGTAGT

10401 TCGCCAGTTA ATAGTTTGCG CAACGTTGTT GCCATTGCTA CAGGCATCGT

10451 GGTGTCACGC TCGTCGTTTG GTATGGCTTC ATTCAGCTCC GGTTCCCAAC

10501 GATCAAGGCG AGTTACATGA TCCCCCATGT TGTGCAAAAA AGCGGTTAGC

10551 TCCTTCGGTC CTCCGATCGT TGTCAGAAGT AAGTTGGCCG CAGTGTTATC

10601 ACTCATGGTT ATGGCAGCAC TGCATAATTC TCTTACTGTC ATGCCATCCG

10651 TAAGATGCTT TTCTGTGACT GGTGAGTACT CAACCAAGTC ATTCTGAGAA

10701 TAGTGTATGC GGCGACCGAG TTGCTCTTGC CCGGCGTCAA TACGGGATAA

10751 TACCGCGCCA CATAGCAGAA CTTTAAAAGT GCTCATCATT GGAAAACGTT

10801 CTTCGGGGCG AAAACTCTCA AGGATCTTAC CGCTGTTGAG ATCCAGTTCG

10851 ATGTAACCCA CTCGTGCACC CAACTGATCT TCAGCATCTT TTACTTTCAC

10901 CAGCGTTTCT GGGTGAGCAA AAACAGGAAG GCAAAATGCC GCAAAAAAGG

10951 GAATAAGGGC GACACGGAAA TGTTGAATAC TCATACTCTT CCTTTTTCAA

11001 TATTATTGAA GCATTTATCA GGGTTATTGT CTCATGAGCG GATACATATT

11051 TGAATGTATT TAGAAAAATA AACAAATAGG GGTTCCGCGC ACATTTCCCC

11101 GAAAAGTGCC ACCTGAC

Features:

2622 – 3276 bp CMV promoter

3348 – 4060 bp TALE N-terminal

4061 – 4071 bp BsmBI

4072 – 6433 bp CmR-ccdB

6427 – 6437 bp BsmBI

6438 – 6970 bp TALE C-terminal

6971 – 7003 bp NLS

7004 – 7174 bp VP64

7181 – 7243 bp 2A

7244 – 7951 bp mCherry

(C) pcDNA6-3A-luciferase:

1 AATTCTGCAG ATATCCAGCA CAGTGGCGGC CGCTCGAGTC TAGAGGGCCC

51 GCGGTTCGAA GGTAAGCCTA TCCCTAACCC TCTCCTCGGT CTCGATTCTA

101 CGCGTACCGG TCATCATCAC CATCACCATT GAGTTTAAAC CCGCTGATCA

151 GCCTCGACTG TGCCTTCTAG TTGCCAGCCA TCTGTTGTTT GCCCCTCCCC

201 CGTGCCTTCC TTGACCCTGG AAGGTGCCAC TCCCACTGTC CTTTCCTAAT

251 AAAATGAGGA AATTGCATCG CATTGTCTGA GTAGGTGTCA TTCTATTCTG

301 GGGGGTGGGG TGGGGCAGGA CAGCAAGGGG GAGGATTGGG AAGACAATAG

351 CAGGCATGCT GGGGATGCGG TGGGCTCTAT GGCTTCTGAG GCGGAAAGAA

401 CCAGCTGGGG CTCTAGGGGG TATCCCCACG CGCCCTGTAG CGGCGCATTA

451 AGCGCGGCGG GTGTGGTGGT TACGCGCAGC GTGACCGCTA CACTTGCCAG

501 CGCCCTAGCG CCCGCTCCTT TCGCTTTCTT CCCTTCCTTT CTCGCCACGT

551 TCGCCGGCTT TCCCCGTCAA GCTCTAAATC GGGGGCTCCC TTTAGGGTTC

601 CGATTTAGTG CTTTACGGCA CCTCGACCCC AAAAAACTTG ATTAGGGTGA

651 TGGTTCACGT AGTGGGCCAT CGCCCTGATA GACGGTTTTT CGCCCTTTGA

701 CGTTGGAGTC CACGTTCTTT AATAGTGGAC TCTTGTTCCA AACTGGAACA

751 ACACTCAACC CTATCTCGGT CTATTCTTTT GATTTATAAG GGATTTTGCC

801 GATTTCGGCC TATTGGTTAA AAAATGAGCT GATTTAACAA AAATTTAACG

851 CGAATTAATT CTGTGGAATG TGTGTCAGTT AGGGTGTGGA AAGTCCCCAG

901 GCTCCCCAGC AGGCAGAAGT ATGCAAAGCA TGCATCTCAA TTAGTCAGCA

951 ACCAGGTGTG GAAAGTCCCC AGGCTCCCCA GCAGGCAGAA GTATGCAAAG

1001 CATGCATCTC AATTAGTCAG CAACCATAGT CCCGCCCCTA ACTCCGCCCA

1051 TCCCGCCCCT AACTCCGCCC AGTTCCGCCC ATTCTCCGCC CCATGGCTGA

1101 CTAATTTTTT TTATTTATGC AGAGGCCGAG GCCGCCTCTG CCTCTGAGCT

1151 ATTCCAGAAG TAGTGAGGAG GCTTTTTTGG AGGCCTAGGC TTTTGCAAAA

1201 AGCTCCCGGG AGCTTGTATA TCCATTTTCG GATCTGATCA GCACACCATG

1251 ACCGAGTACA AGCCCACGGT GCGCCTCGCC ACCCGCGACG ACGTCCCCAG

1301 GGCCGTACGC ACCCTCGCCG CCGCGTTCGC CGACTACCCC GCCACGCGCC

1351 ACACCGTCGA TCCGGACCGC CACATCGAGC GGGTCACCGA GCTGCAAGAA

1401 CTCTTCCTCA CGCGCGTCGG GCTCGACATC GGCAAGGTGT GGGTCGCGGA

1451 CGACGGCGCC GCGGTGGCGG TCTGGACCAC GCCGGAGAGC GTCGAAGCGG

1501 GGGCGGTGTT CGCCGAGATC GGCCCGCGCA TGGCCGAGTT GAGCGGTTCC

1551 CGGCTGGCCG CGCAGCAACA GATGGAAGGC CTCCTGGCGC CGCACCGGCC

1601 CAAGGAGCCC GCGTGGTTCC TGGCCACCGT CGGCGTCTCG CCCGACCACC

1651 AGGGCAAGGG TCTGGGCAGC GCCGTCGTGC TCCCCGGAGT GGAGGCGGCC

1701 GAGCGCGCCG GGGTGCCCGC CTTCCTGGAG ACCTCCGCGC CCCGCAACCT

1751 CCCCTTCTAC GAGCGGCTCG GCTTCACCGT CACCGCCGAC GTCGAGTGCC

1801 CGAAGGACCG CGCGACCTGG TGCATGACCC GCAAGCCCGG TGCCTGACGC

1851 CCGGTGCTAC GAGATTTCGA TTCCACCGCC GCCTTCTATG AAAGGTTGGG

1901 CTTCGGAATC GTTTTCCGGG ACGCCGGCTG GATGATCCTC CAGCGCGGGG

1951 ATCTCATGCT GGAGTTCTTC GCCCACCCCA ACTTGTTTAT TGCAGCTTAT

2001 AATGGTTACA AATAAAGCAA TAGCATCACA AATTTCACAA ATAAAGCATT

2051 TTTTTCACTG CATTCTAGTT GTGGTTTGTC CAAACTCATC AATGTATCTT

2101 ATCATGTCTG TATACCGTCG ACCTCTAGCT AGAGCTTGGC GTAATCATGG

2151 TCATAGCTGT TTCCTGTGTG AAATTGTTAT CCGCTCACAA TTCCACACAA

2201 CATACGAGCC GGAAGCATAA AGTGTAAAGC CTGGGGTGCC TAATGAGTGA

2251 GCTAACTCAC ATTAATTGCG TTGCGCTCAC TGCCCGCTTT CCAGTCGGGA

2301 AACCTGTCGT GCCAGCTGCA TTAATGAATC GGCCAACGCG CGGGGAGAGG

2351 CGGTTTGCGT ATTGGGCGCT CTTCCGCTTC CTCGCTCACT GACTCGCTGC

2401 GCTCGGTCGT TCGGCTGCGG CGAGCGGTAT CAGCTCACTC AAAGGCGGTA

2451 ATACGGTTAT CCACAGAATC AGGGGATAAC GCAGGAAAGA ACATGTGAGC

2501 AAAAGGCCAG CAAAAGGCCA GGAACCGTAA AAAGGCCGCG TTGCTGGCGT

2551 TTTTCCATAG GCTCCGCCCC CCTGACGAGC ATCACAAAAA TCGACGCTCA

2601 AGTCAGAGGT GGCGAAACCC GACAGGACTA TAAAGATACC AGGCGTTTCC

2651 CCCTGGAAGC TCCCTCGTGC GCTCTCCTGT TCCGACCCTG CCGCTTACCG

2701 GATACCTGTC CGCCTTTCTC CCTTCGGGAA GCGTGGCGCT TTCTCATAGC

2751 TCACGCTGTA GGTATCTCAG TTCGGTGTAG GTCGTTCGCT CCAAGCTGGG

2801 CTGTGTGCAC GAACCCCCCG TTCAGCCCGA CCGCTGCGCC TTATCCGGTA

2851 ACTATCGTCT TGAGTCCAAC CCGGTAAGAC ACGACTTATC GCCACTGGCA

2901 GCAGCCACTG GTAACAGGAT TAGCAGAGCG AGGTATGTAG GCGGTGCTAC

2951 AGAGTTCTTG AAGTGGTGGC CTAACTACGG CTACACTAGA AGAACAGTAT

3001 TTGGTATCTG CGCTCTGCTG AAGCCAGTTA CCTTCGGAAA AAGAGTTGGT

3051 AGCTCTTGAT CCGGCAAACA AACCACCGCT GGTAGCGGTG GTTTTTTTGT

3101 TTGCAAGCAG CAGATTACGC GCAGAAAAAA AGGATCTCAA GAAGATCCTT

3151 TGATCTTTTC TACGGGGTCT GACGCTCAGT GGAACGAAAA CTCACGTTAA

3201 GGGATTTTGG TCATGAGATT ATCAAAAAGG ATCTTCACCT AGATCCTTTT

3251 AAATTAAAAA TGAAGTTTTA AATCAATCTA AAGTATATAT GAGTAAACTT

3301 GGTCTGACAG TTACCAATGC TTAATCAGTG AGGCACCTAT CTCAGCGATC

3351 TGTCTATTTC GTTCATCCAT AGTTGCCTGA CTCCCCGTCG TGTAGATAAC

3401 TACGATACGG GAGGGCTTAC CATCTGGCCC CAGTGCTGCA ATGATACCGC

3451 GAGACCCACG CTCACCGGCT CCAGATTTAT CAGCAATAAA CCAGCCAGCC

3501 GGAAGGGCCG AGCGCAGAAG TGGTCCTGCA ACTTTATCCG CCTCCATCCA

3551 GTCTATTAAT TGTTGCCGGG AAGCTAGAGT AAGTAGTTCG CCAGTTAATA

3601 GTTTGCGCAA CGTTGTTGCC ATTGCTACAG GCATCGTGGT GTCACGCTCG

3651 TCGTTTGGTA TGGCTTCATT CAGCTCCGGT TCCCAACGAT CAAGGCGAGT

3701 TACATGATCC CCCATGTTGT GCAAAAAAGC GGTTAGCTCC TTCGGTCCTC

3751 CGATCGTTGT CAGAAGTAAG TTGGCCGCAG TGTTATCACT CATGGTTATG

3801 GCAGCACTGC ATAATTCTCT TACTGTCATG CCATCCGTAA GATGCTTTTC

3851 TGTGACTGGT GAGTACTCAA CCAAGTCATT CTGAGAATAG TGTATGCGGC

3901 GACCGAGTTG CTCTTGCCCG GCGTCAATAC GGGATAATAC CGCGCCACAT

3951 AGCAGAACTT TAAAAGTGCT CATCATTGGA AAACGTTCTT CGGGGCGAAA

4001 ACTCTCAAGG ATCTTACCGC TGTTGAGATC CAGTTCGATG TAACCCACTC

4051 GTGCACCCAA CTGATCTTCA GCATCTTTTA CTTTCACCAG CGTTTCTGGG

4101 TGAGCAAAAA CAGGAAGGCA AAATGCCGCA AAAAAGGGAA TAAGGGCGAC

4151 ACGGAAATGT TGAATACTCA TACTCTTCCT TTTTCAATAT TATTGAAGCA

4201 TTTATCAGGG TTATTGTCTC ATGAGCGGAT ACATATTTGA ATGTATTTAG

4251 AAAAATAAAC AAATAGGGGT TCCGCGCACA TTTCCCCGAA AAGTGCCACC

4301 TGACGTCGAC GGATCGGGAG ATCTCCCGAT CCCCTATGGT GCACTCTCAG

4351 TACAATCTGC TCTGATGCCG CATAGTTAAG CCAGTATCTG CTCCCTGCTT

4401 GTGTGTTGGA GGTCGCTGAG TAGTGCGCGA GCAAAATTTA AGCTACAACA

4451 AGGCAAGGCT TGACCGACAA TTGCATGAAG AATCTGCTTA GGGTTAGGCG

4501 TTTTGCGCTG CTTCGCGATG TACGGGCCAG ATATACGCGT TACTGGAGCC

4551 ATCTGGCCAA ATACGTAGGC GTGTACGGTG GGAGGCCTAT ATAAGCAGAG

4601 CTCGTTTAGT GAACCGTCAG ATCGCCTGGA GGTACATGGA AGACGCCAAA

4651 AACATAAAGA AAGGCCCGGC GCCATTCTAT CCGCTGGAAG ATGGAACCGC

4701 TGGAGAGCAA CTGCATAAGG CTATGAAGAG ATACGCCCTG GTTCCTGGAA

4751 CAATTGCTTT TACAGATGCA CATATCGAGG TGGACATCAC TTACGCTGAG

4801 TACTTCGAAA TGTCCGTTCG GTTGGCAGAA GCTATGAAAC GATATGGGCT

4851 GAATACAAAT CACAGAATCG TCGTATGCAG TGAAAACTCT CTTCAATTCT

4901 TTATGCCGGT GTTGGGCGCG TTATTTATCG GAGTTGCAGT TGCGCCCGCG

4951 AACGACATTT ATAATGAACG TGAATTGCTC AACAGTATGG GCATTTCGCA

5001 GCCTACCGTG GTGTTCGTTT CCAAAAAGGG GTTGCAAAAA ATTTTGAACG

5051 TGCAAAAAAA GCTCCCAATC ATCCAAAAAA TTATTATCAT GGATTCTAAA

5101 ACGGATTACC AGGGATTTCA GTCGATGTAC ACGTTCGTCA CATCTCATCT

5151 ACCTCCCGGT TTTAATGAAT ACGATTTTGT GCCAGAGTCC TTCGATAGGG

5201 ACAAGACAAT TGCACTGATC ATGAACTCCT CTGGATCTAC TGGTCTGCCT

5251 AAAGGTGTCG CTCTGCCTCA TAGAACTGCC TGCGTGAGAT TCTCGCATGC

5301 CAGAGATCCT ATTTTTGGCA ATCAAATCAT TCCGGATACT GCGATTTTAA

5351 GTGTTGTTCC ATTCCATCAC GGTTTTGGAA TGTTTACTAC ACTCGGATAT

5401 TTGATATGTG GATTTCGAGT CGTCTTAATG TATAGATTTG AAGAAGAGCT

5451 GTTTCTGAGG AGCCTTCAGG ATTACAAGAT TCAAAGTGCG CTGCTGGTGC

5501 CAACCCTATT CTCCTTCTTC GCCAAAAGCA CTCTGATTGA CAAATACGAT

5551 TTATCTAATT TACACGAAAT TGCTTCTGGT GGCGCTCCCC TCTCTAAGGA

5601 AGTCGGGGAA GCGGTTGCCA AGAGGTTCCA TCTGCCAGGT ATCAGGCAAG

5651 GATATGGGCT CACTGAGACT ACATCAGCTA TTCTGATTAC ACCCGAGGGG

5701 GATGATAAAC CGGGCGCGGT CGGTAAAGTT GTTCCATTTT TTGAAGCGAA

5751 GGTTGTGGAT CTGGATACCG GGAAAACGCT GGGCGTTAAT CAAAGAGGCG

5801 AACTGTGTGT GAGAGGTCCT ATGATTATGT CCGGTTATGT AAACAATCCG

5851 GAAGCGACCA ACGCCTTGAT TGACAAGGAT GGATGGCTAC ATTCTGGAGA

5901 CATAGCTTAC TGGGACGAAG ACGAACACTT CTTCATCGTT GACCGCCTGA

5951 AGTCTCTGAT TAAGTACAAA GGCTATCAGG TGGCTCCCGC TGAATTGGAA

6001 TCCATCTTGC TCCAACACCC CAACATCTTC GACGCAGGTG TCGCAGGTCT

6051 TCCCGACGAT GACGCCGGTG AACTTCCCGC CGCCGTTGTT GTTTTGGAGC

6101 ACGGAAAGAC GATGACGGAA AAAGAGATCG TGGATTACGT CGCCAGTCAA

```
6151 GTAACAACCG CGAAAAAGTT GCGCGGAGGA GTTGTGTTTG TGGACGAAGT

6201 ACCGAAAGGT CTTACCGGAA AACTCGACGC AAGAAAAATC AGAGAGATCC

6251 TCATAAAGGC CAAGAAGGGC GGAAAGATCG CCGTGTAAG
```

Features:

4553 – 4567 bp TALE binding site

4567 – 4635 bp miniCMV promoter

4636 – 6288 bp firefly luciferase
