# Supplementary material for: ULtiMATE System for Rapid Assembly of Customized TAL Effectors
Source: PLoS One. 2013 Sep 27;8(9):e75649. doi: 10.1371/journal.pone.0075649 (PMC3815405; doi:10.1371/journal.pone.0075649)

Supporting information, Figure S1

Figure S1. Example of PCR design for ULtiMATE system using the JAVA program.

(Before using this software, please read the “readme.txt” file for computer settings.)

Step 1. Open the software by double click the file "ULtiMATE_PCR_Design_for_Windows.bat” in PC or run "ULtiMATE_PCR_Design.jar" using the Jar Launcher (right click and choose "Jar Launcher" to open it) in Mac or Linux.

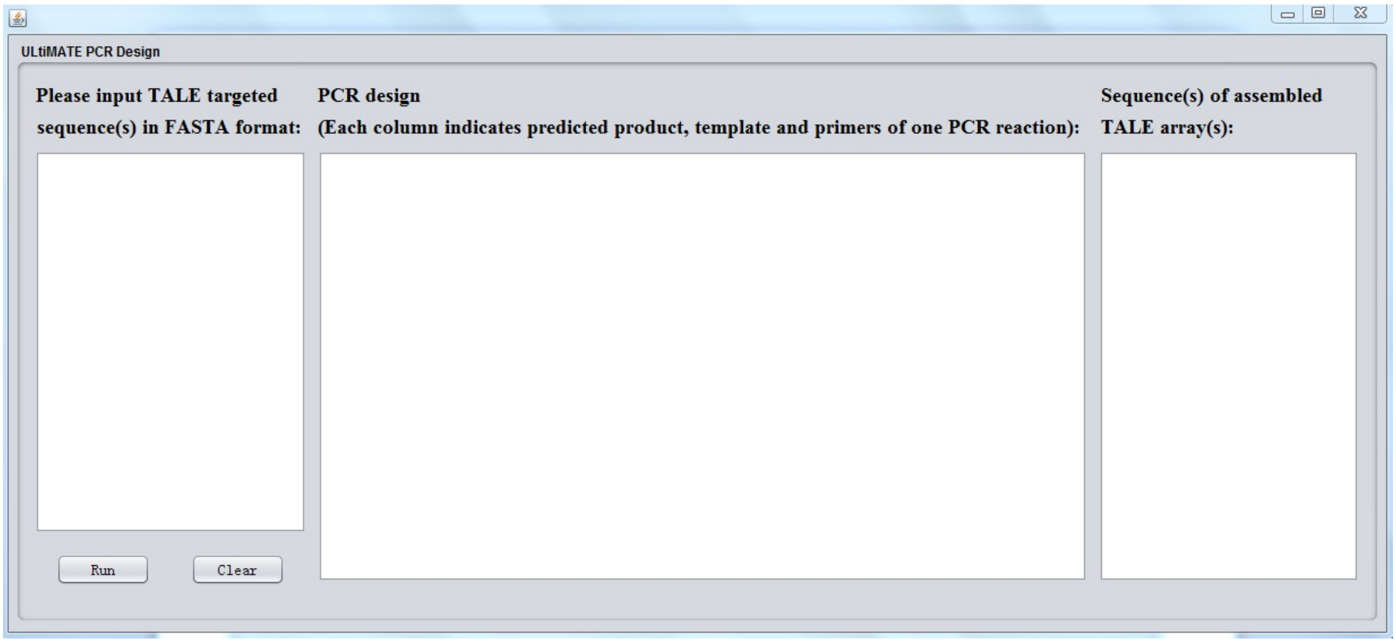

Step 2. Insert target sequence(s) (in FASTA format) of TALEN(s)/TALE-TA(s) in left window.

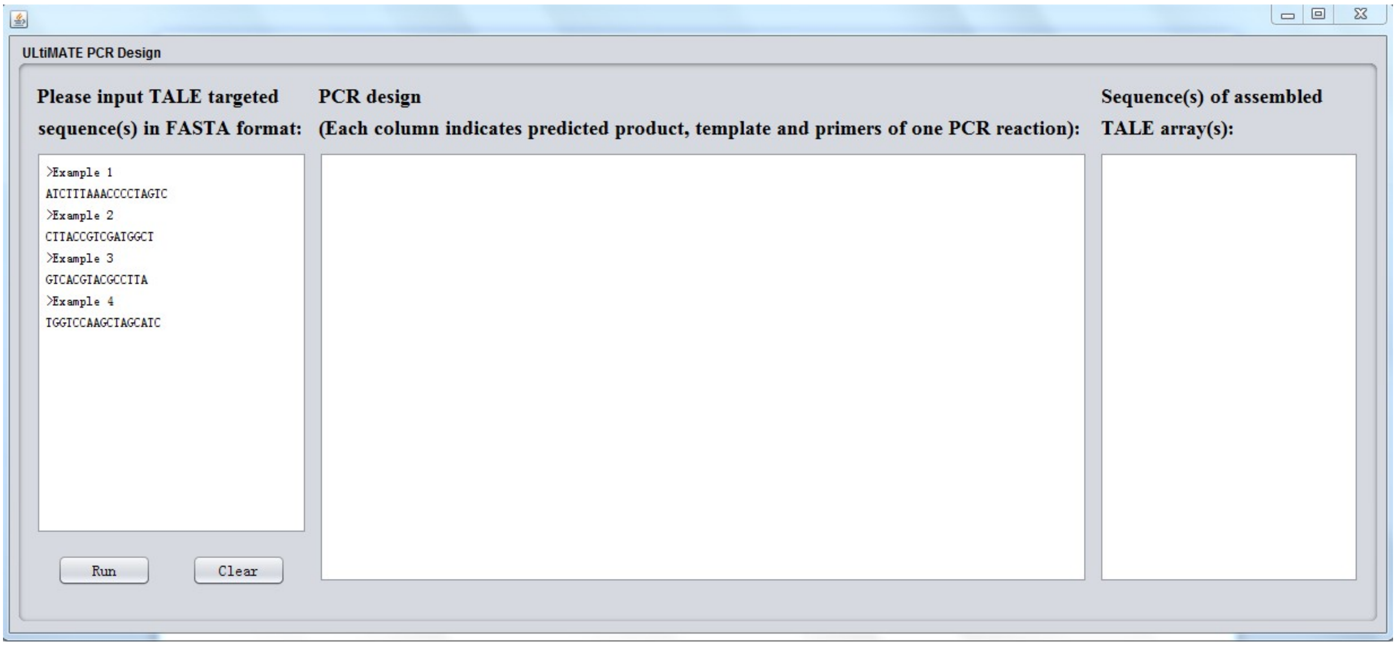

Step3. Click “Run” to acquire the output. Templates and primers for PCR amplification are shown in the middle window, and sequence(s) of the final assembled TALE array(s) are shown in the right window.

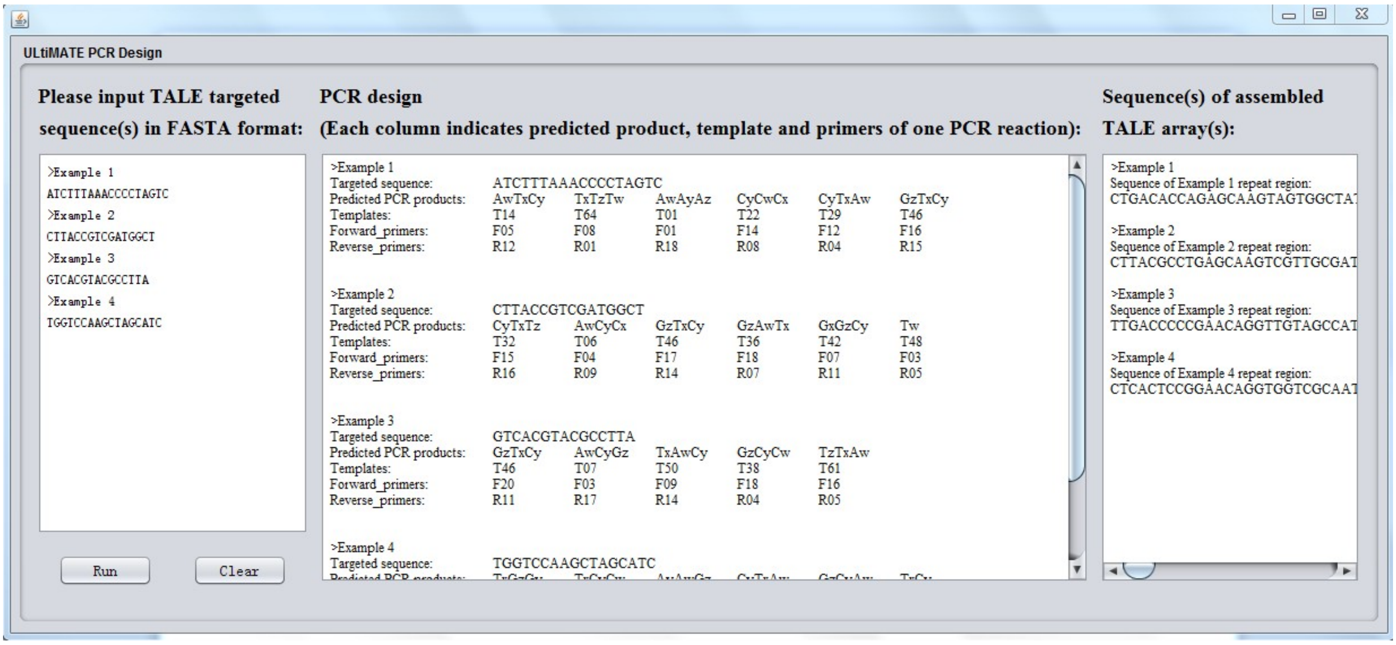

Supplement: Figure S1 — Example of PCR design for ULtiMATE system using the JAVA program. (PDF) [file pone.0075649.s002.pdf]
