# Supplementary material for: ULtiMATE System for Rapid Assembly of Customized TAL Effectors
Source: PLoS One. 2013 Sep 27;8(9):e75649. doi: 10.1371/journal.pone.0075649 (PMC3815405; doi:10.1371/journal.pone.0075649)

ATG5: Indels (59.8%)

TALEN^L (ATG5) TALEN^R (ATG5)

GTTTCACGCTATATCAGGATGAGATAACTGAAAGGGAAGCAGAACCAT

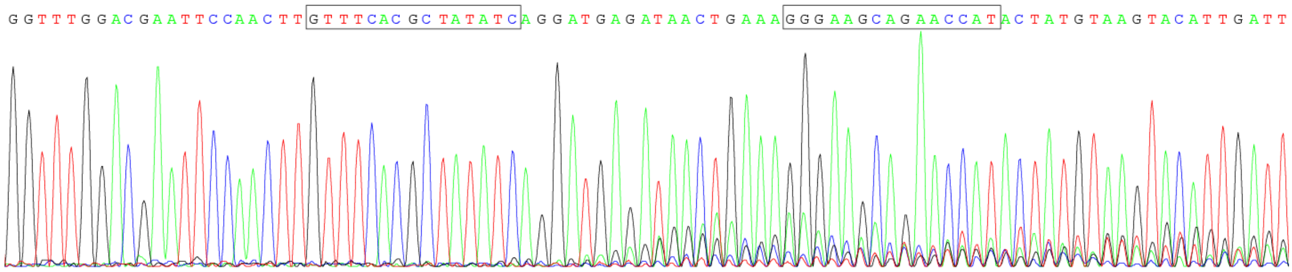

HBEGF: Indels (58.6%)

TALEN^L (HBEGF) TALEN^R (HBEGF)

CCCACTGTATCCACGGACCAGCTGCTACCCCTAGGAGGCGGCCGGG

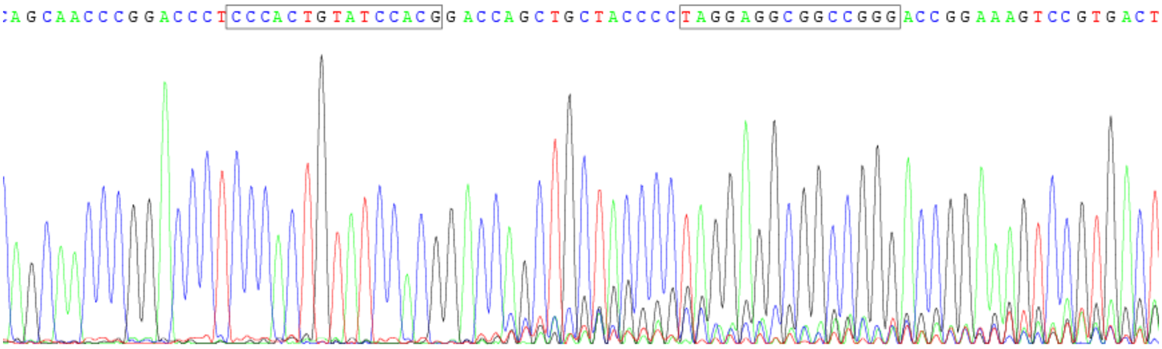

HSP90AB1: Indels (28.9%)

TALEN^L (HSP90AB1) TALEN^R (HSP90AB1)

ACCTTCTATTCCAACAAGGAGATTTTCCTTCGGGAGTTGATCTCT

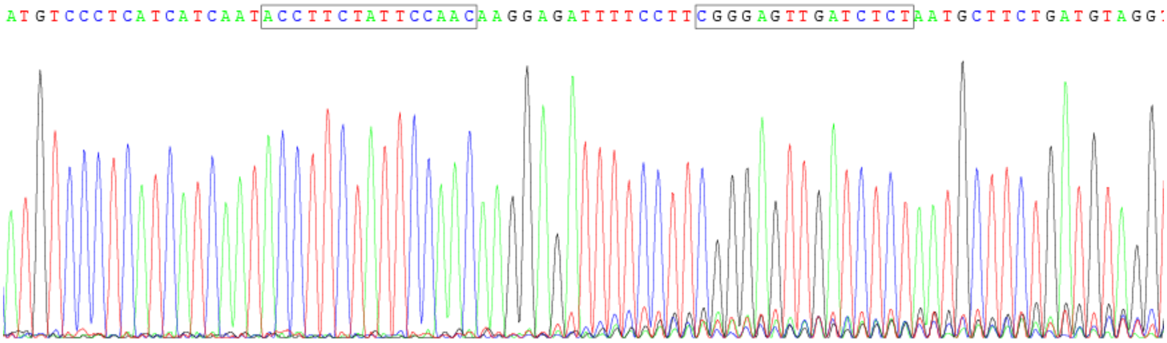

LRP1: Indels (70.1%)

TALEN^L (LRP1) TALEN^R (LRP1)

AAGACTTGCAGCCCCAAGCAGTTTGCCTGCAGAGATCAAATAACCTGT

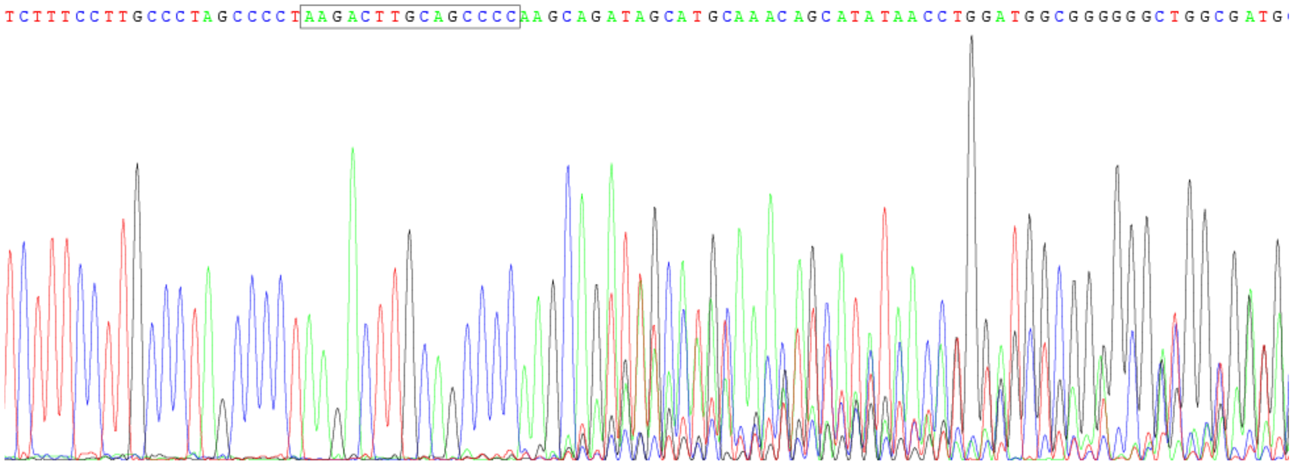

PLXNA2: Indels (39.1%)

TALEN^{L} (PLXNA2) TALEN^{R} (PLXNA2)

TTGACATCTTCTACATCTACGGCTTTGCTAGTGGGGGCTTTGTCT

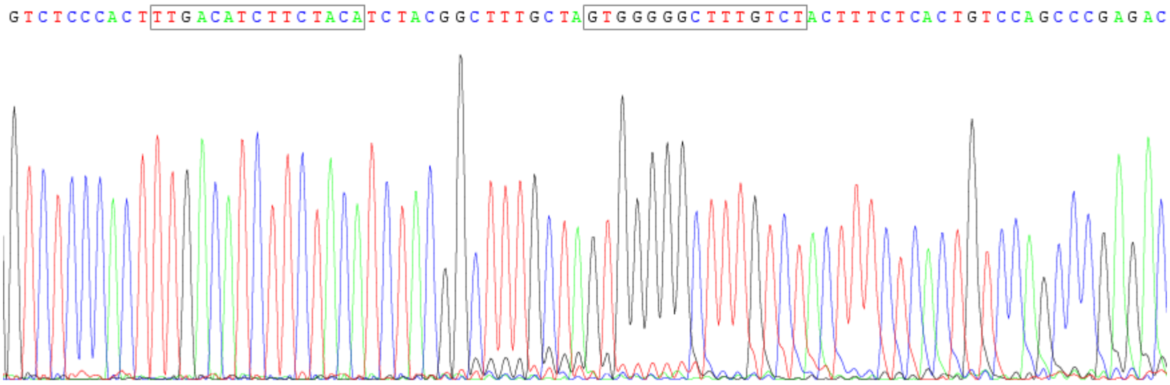

VPS15: Indels (26.0%)

TALEN^{L} (VPS15) TALEN^{R} (VPS15)

TCAACTCCGCTTGTAGACTTAAATAGCAATCAGAGAACAAGAGGAG

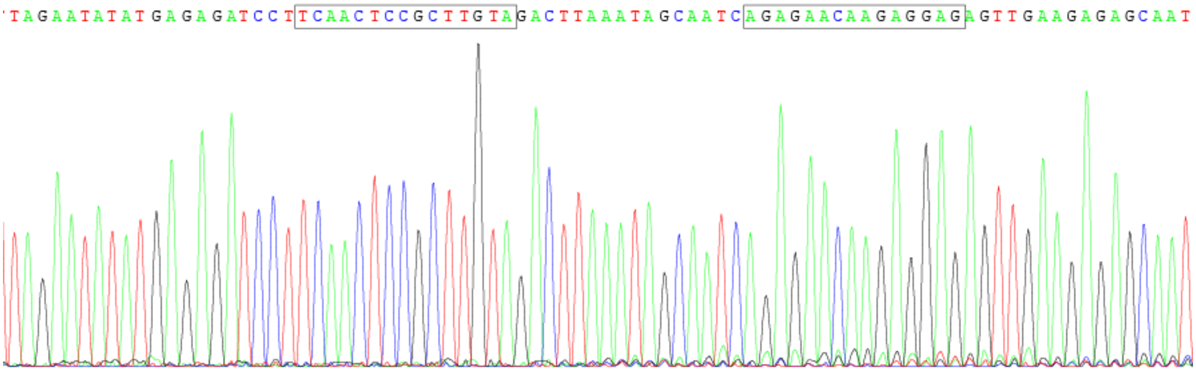

VPS34: Indels (36.0%)

TALEN^{L} (VPS34) TALEN^{R} (VPS34)

CTTAAGCTGGACGTTGATATCCAGGTCACAACTATAGATGTAGTGAA

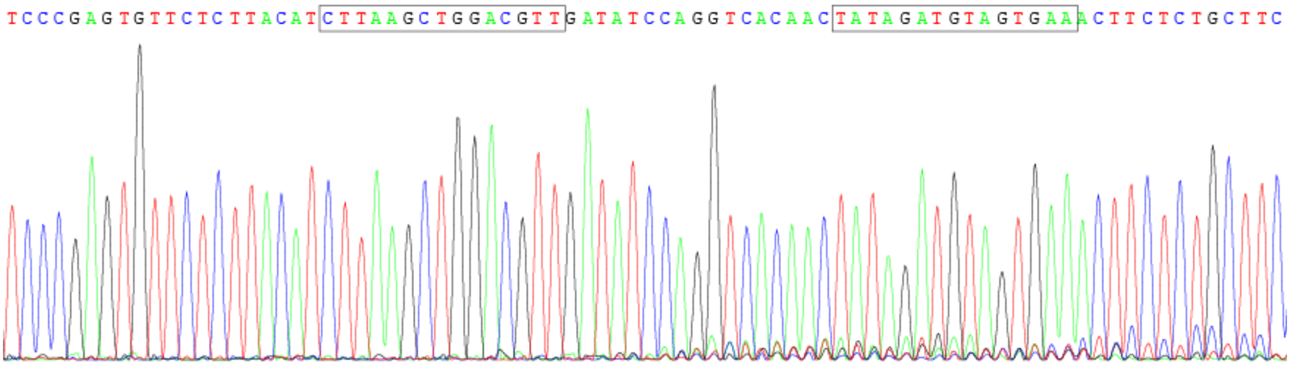

Supplement: Figure S2 — TALENs’ effects on patterns of sequencing chromatogram of targeted regions. Partial sequences of seven representative genes (ATG5, HBEGF, HSP90AB1, LRP1, PLXNA2, VPS15, and VPS34) in TALENs targeting regions (underlined are binding sequences for TALENL and TALENR) are indicated by the four-color sequencing chromatogram. The baseline noises indicate the occurrence of indels. The percentage of NHEJ induced indels was assayed using the mismatch-sensitive T7E1 endonuclease (Text S1) and quantified by ImageJ (http://rsbweb.nih.gov/ij/). (PDF) [file pone.0075649.s003.pdf]
