# Supplementary material for: ULtiMATE System for Rapid Assembly of Customized TAL Effectors
Source: PLoS One. 2013 Sep 27;8(9):e75649. doi: 10.1371/journal.pone.0075649 (PMC3815405; doi:10.1371/journal.pone.0075649)
Supplement: Methods S1 — (DOCX) [file pone.0075649.s004.docx]

**Methods S1: TALE cloning protocol for a single TALE array**

1. Based on targeting sequence, select primers and templates for each PCR reaction, following a simple rule that any chosen primer can only be used once. Alternatively, use the attached JAVA program (Software S1) to generate the whole PCR design as well as the sequence of the final TALE array.
2. PCR amplification:

Trimer template (1 ng/μl) 3 μl

Forward primer (1 mM) 3 μl

Reverse primer (1 mM) 3 μl

PfuTurbo Cx DNA Polymerase (2.5 units/μl) 0.15 μl

10 × PfuTurbo Cx Buffer 1.5 μl

dNTP (2.5 mM each) 1.5 μl

ddH_2_O up to 15 μl

PCR program:

95^o^C 3 min

95^o^C 30 sec \

60^o^C 30 sec | 30 cycles

72^o^C 30 sec /

72^o^C 10 min
4^o^C Soaking

1. Combine all PCR products into one tube, and proceed with the USER^TM^ digestion plus ligation sequentially as follows:

PCR products 50 ~ 70 μl

USER^TM^ enzyme (1 unit/μl) 1 μl

37^o^C, 15 min

T4 DNA ligase (400 cohesive end units/μl) 1 μl

10 mM ATP 5 ~ 7 μl

37^o^C, 30 min

4. Gel-purification of the ligated DNA fragments from 2% agarose gel. Extract the DNA band based on the expected size of TALE array (the top band).

5. Ligation of assembled TALE repeats into cloning vectors by cycle of BsmBI digestion and ligation:

TALE fragments 1 μl

BsmBI (Esp3I) (10 units/μl) 0.75 μl

T4 DNA ligase (400 cohesive end units/μl) 0.25 μl

10 mM ATP 1 μl

DTT (10 mM) 1 μl

10 x Tango buffer 1 μl

Plasmid (TALE expression vector) 20 ng

ddH_2_O up to 10 μl

Reaction program:

37^o^C, 5 min \

| 6 cycles

25^o^C, 5 min /

4^o^C, soaking

1. 5 μl products were transformed into Trans1-T1 competent cells, and plated onto solid LB medium with 100 μg/ml ampicillin.
