# Supplementary material for: ULtiMATE System for Rapid Assembly of Customized TAL Effectors
Source: PLoS One. 2013 Sep 27;8(9):e75649. doi: 10.1371/journal.pone.0075649 (PMC3815405; doi:10.1371/journal.pone.0075649)
Supplement: Table S1 — (PDF) [file pone.0075649.s006.pdf]

Supporting information, Table S1

Table S1 Template preparation for PCR amplification of customized TAL effector repeat units

A. Design of 4 types of basic modules encoding the same TAL effector repeat units

W type module:

5’ CTGACACCAGAGCAAGTAGTGGCTATTGCAAGTNNNNNNGGTGGCAAACAAGCGCTGGAGACCGTGCAGAGGCTCCTTCCGGTGCTCTGCCAAGCACACGGT
L T P E Q V V A I A S X X G G K Q A L E T V Q R L L P V L C Q A H G

X type module:

5’ CTCACTCCGGAACAGGTGGTCGCAATCGCGAGCNNNNNNGGCGGCAAGCAAGCCCTTGAGACAGTCCAAAGACTTTTGCCTGTCCTTTGTCAGGCGCATGGC
L T P E Q V V A I A S X X G G K Q A L E T V Q R L L P V L C Q A H G

Y type module:

5’ CTTACGCCTGAGCAAGTCGTTGCGATCGCCTCCNNNNNNGGCGGAAAACAGGCTTTGGAAACCGTGCAGCGGTTGCTGCCCGTTTTGTGCCAAGCCCACGGA
L T P E Q V V A I A S X X G G K Q A L E T V Q R L L P V L C Q A H G

Z type module:

5’ TTGACCCCCGAACAGGTTGTAGCCATAGCTTCTNNNNNNGGAGGTAAGCAGGCACTGGAAACCGTGCAGCGCCTGCTCCCAGTACTGTGTCAGGCTCATGGG
L T P E Q V V A I A S X X G G K Q A L E T V Q R L L P V L C Q A H G

B. Archive of 12 TAL effector repeat units (1-mers)

	W Type	X Type	Y Type	Z Type
**A-targeting Module (RVDs = NI)** (NNNNNN = AACATC)	A^w		A^y	A^z
**T-targeting Module (RVDs = NG)** (NNNNNN = AACGGC)	T^w	T^x		T^z
**C-targeting Module (RVDs = HD)** (NNNNNN = CACGAC)	C^w	C^x	C^y	
**G-targeting Module (RVDs = NN)** (NNNNNN = AACAAC)		G^x	G^y	G^z

C. Archive of 64 pre-assembled TALE repeat units (3-mers)

<table>
<tr><td>T01 | $A^w A^y A^z$</td><td>T09 | $A^w G^z A^y$</td><td>T17 | $C^y A^w A^z$</td><td>T25 | $C^y G^z A^w$</td><td>T33 | $G^z A^w A^y$</td><td>T41 | $G^x G^z A^w$</td><td>T49 | $T^x A^w A^y$</td><td>T57 | $T^x G^z A^w$</td></tr>
<tr><td>T02 | $A^z A^w C^y$</td><td>T10 | $A^w G^z C^y$</td><td>T18 | $C^y A^w C^x$</td><td>T26 | $C^y G^z C^x$</td><td>T34 | $G^z A^w C^y$</td><td>T42 | $G^x G^z C^y$</td><td>T50 | $T^x A^w C^y$</td><td>T58 | $T^x G^z C^y$</td></tr>
<tr><td>T03 | $A^y A^w G^z$</td><td>T11 | $A^w G^z G^x$</td><td>T19 | $C^y A^w G^z$</td><td>T27 | $C^y G^z G^x$</td><td>T35 | $G^z A^w G^y$</td><td>T43 | $G^z G^x G^y$</td><td>T51 | $T^x A^w G^z$</td><td>T59 | $T^x G^z G^y$</td></tr>
<tr><td>T04 | $A^y A^w T^x$</td><td>T12 | $A^w G^z T^x$</td><td>T20 | $C^y A^w T^x$</td><td>T28 | $C^y G^z T^x$</td><td>T36 | $G^z A^w T^x$</td><td>T44 | $G^y G^z T^x$</td><td>T52 | $T^x A^w T^z$</td><td>T60 | $T^x G^z T^w$</td></tr>
<tr><td>T05 | $A^w C^y A^z$</td><td>T13 | $A^w T^x A^z$</td><td>T21 | $C^x C^y A^w$</td><td>T29 | $C^y T^x A^w$</td><td>T37 | $G^z C^y A^w$</td><td>T45 | $G^z T^x A^w$</td><td>T53 | $T^x C^y A^w$</td><td>T61 | $T^z T^x A^w$</td></tr>
<tr><td>T06 | $A^w C^y C^x$</td><td>T14 | $A^w T^x C^y$</td><td>T22 | $C^y C^w C^x$</td><td>T30 | $C^y T^x C^w$</td><td>T38 | $G^z C^y C^w$</td><td>T46 | $G^z T^x C^y$</td><td>T54 | $T^x C^y C^w$</td><td>T62 | $T^z T^x C^y$</td></tr>
<tr><td>T07 | $A^w C^y G^z$</td><td>T15 | $A^w T^x G^z$</td><td>T23 | $C^w C^y G^z$</td><td>T31 | $C^y T^x G^z$</td><td>T39 | $G^z C^y G^x$</td><td>T47 | $G^z T^x G^y$</td><td>T55 | $T^x C^y G^z$</td><td>T63 | $T^w T^x G^z$</td></tr>
<tr><td>T08 | $A^w C^y T^x$</td><td>T16 | $A^w T^x T^z$</td><td>T24 | $C^w C^y T^x$</td><td>T32 | $C^y T^x T^z$</td><td>T40 | $G^z C^y T^x$</td><td>T48 | $G^z T^x T^w$</td><td>T56 | $T^x C^y T^w$</td><td>T64 | $T^x T^z T^w$</td></tr>
</table>

The short and full names of each template are separated by the symbol |.
