# Supplementary material for: ULtiMATE System for Rapid Assembly of Customized TAL Effectors
Source: PLoS One. 2013 Sep 27;8(9):e75649. doi: 10.1371/journal.pone.0075649 (PMC3815405; doi:10.1371/journal.pone.0075649)
Supplement: Table S2 — (PDF) [file pone.0075649.s007.pdf]

Supporting information, Table S2

Table S2 Uracil primers used for PCR amplification of customized TALE repeats

A. Primer matrix (40) for PCR amplification of modules

Upstream Module	Forward Primer				Reverse Primer				Downstream Module
	Module W	Module X	Module Y	Module Z	Module W	Module X	Module Y	Module Z	
Module W	F01 | F-Ww	F06 | F-Xw	F11 | F-Yw	F16 | F-Zw	R01 | R-Ww	R06 | R-Xw	R11 | R-Yw	R16 | R-Zw	Module W
Module X	F02 | F-Wx	F07 | F-Xx	F12 | F-Yx	F17 | F-Zx	R02 | R-Wx	R07 | R-Xx	R12 | R-Yx	R17 | R-Zx	Module X
Module Y	F03 | F-Wy	F08 | F-Xy	F13 | F-Yy	F18 | F-Zy	R03 | R-Wy	R08 | R-Xy	R13 | R-Yy	R18 | R-Zy	Module Y
Module Z	F04 | F-Wz	F09 | F-Xz	F14 | F-Yz	F19 | F-Zz	R04 | R-Wz	R09 | R-Xz	R14 | R-Yz	R19 | R-Zz	Module Z
N-terminal Backbone	F05 | F-W5	F10 | F-X5	F15 | F-Y5	F20 | F-Z5	R05 | R-W3	R10 | R-X3	R15 | R-Y3	R20 | R-Z3	C-terminal Backbone

The short and full names of each primer are separated by the symbol |.

B. Primer sequences listed by compatibility

Reverse Primer Used for Upstream Module Amplification		Sticky End Sequences of Adjacent Modules after USER™ or BsmBI Digestion	Forward Primer Used for Downstream Module Amplification	
Name	Sequence		Sequence	Name
R01 | R-Ww	5’- AGACCGUGTGCTTGGCAGAGCACCGGA	---ACGGTCT 3’<br>3’ TGCCAGA---	5’- ACGGTCUGACACCAGAGCAAGTAGTGGCTATTG	F01 | F-Ww
R02 | R-Wx	5’- ACCGTGUGCTTGGCAGAGCACCGGAAGG	---ACACGGT 3’<br>3’ TGTGCCA---	5’- ACACGGUCTCACTCCGGAACAGGTGGTCGCA	F06 | F-Xw
R03 | R-Wy	5’- AAGACCGTGUGCTTGGCAGAGCACCGGAAGG	---ACACGGTCTT 3’<br>3’ TGTGCCAGAA---	5’- ACACGGTCTUACGCCTGAGCAAGTCGTTGCG	F11 | F-Yw
R04 | R-Wz	5’- AAACCGTGUGCTTGGCAGAGCACCGGAAGG	---ACACGGTTT 3’<br>3’ TGTGCCAAA---	5’- ACACGGTTUGACCCCCGAACAGGTTGTAGCC	F16 | F-Zw
R06 | R-Xw	5’- AGGCCAUGCGCCTGACAAAGGACAGGCAA	---ATGGCCT 3’<br>3’ TACCGGA---	5’- ATGGCCUGACACCAGAGCAAGTAGTGGCTATTG	F02 | F-Wx
R07 | R-Xx	5’- ATGCGCCUGACAAAGGACAGGCAAAAGTCTTTGGACT	---AGGCGCAT 3’<br>3’ TCCGCGTA---	5’- AGGCGCAUGGCCTCACTCCGGAACAGGTGGTCGCA	F07 | F-Xx
R08 | R-Xy	5’- ACGACTTGCUCAGGCGTAAGGCCATGCGCCTGACAAAGGACA	---AGCAAGTCGT 3’<br>3’ TCGTTCAGCA---	5’- AGCAAGTCGUTGCGATCGCCTCC	F12 | F-Yx
R09 | R-Xz	5’- ACCTGTUCGGGGGTCAAGCCATGCGCCTGACAAAGGACA	---AACAGGT 3’<br>3’ TTGTCCA---	5’- AACAGGUTGTAGCCATAGCTTCT	F17 | F-Zx
R11 | R-Yw	5’- ACTTGCUCTGGTGTCAGTCCGTGGGCTTGGCACAAAACGG	---AGCAAGT 3’<br>3’ TCGTTCA---	5’- AGCAAGUAGTGGCTATTGCAAG	F03 | F-Wy
R12 | R-Yx	5’- AGTGAGUCCGTGGGCTTGGCACAAAACGG	---ACTCACT 3’<br>3’ TGAGTGA---	5’- ACTCACUCCGGAACAGGTGGTCGCAATCG	F08 | F-Xy
R13 | R-Yy	5’- AAGTCCGUGGGCTTGGCACAAAACGGGC	---ACGGACTT 3’<br>3’ TGCCTGAA---	5’- ACGGACTUACGCCTGAGCAAGTCGTTGCGATC	F13 | F-Yy
R14 | R-Yz	5’- AATCCGUGGGCTTGGCACAAAACGGGC	---ACGGATT 3’<br>3’ TGCCTAA---	5’- ACGGATUGACCCCCGAACAGGTTGTAGCC	F18 | F-Zy
R16 | R-Zw	5’- ACTTGCTCUGGTGTCAGCCCATGAGCCTGACACAGTACTGG	---AGAGCAAGT 3’<br>3’ TCTCGTTCA---	5’- AGAGCAAGUAGTGGCTATTGCAAG	F04 | F-Wz
R17 | R-Zx	5’- AGTGAGCCCAUGAGCCTGACACAGTACTGGGAGCA	---ATGGGCTCACT 3’<br>3’ TACCCGAGTGA---	5’- ATGGGCTCACUCCGGAACAGGTGGTCGCAATCG	F09 | F-Xz
R18 | R-Zy	5’- AAGCCCAUGAGCCTGACACAGTACTGGGAGCA	---ATGGGCTT 3’<br>3’ TACCCGAA---	5’- ATGGGCTUACGCCTGAGCAAGTCGTTGCG	F14 | F-Yz
R19 | R-Zz	5’- ACAACCTGUTCGGGGGTCAACCCATGAGCCTGACACAGTACTGGGAGC	---ACAGGTTGT 3’<br>3’ TGTCCAACA---	5’- ACAGGTTGUAGCCATAGCTTCT	F19 | F-Zz
	N-terminal Backbone (BsmBI site)	---GAAC 3’<br>3’ CTTG---	5’- tataCGTCTCaGAACCTGACACCAGAGCAAGTAGTGGCTATTG	F05 | F-W5
			5’- tataCGTCTCaGAACCTCACTCCGGAACAGGTGGTCG	F10 | F-X5
			5’- tataCGTCTCaGAACCTTACGCCTGAGCAAGTCGTTGCG	F15 | F-Y5
			5’- tataCGTCTCaGAACTTGACCCCCGAACAGGTTGTAGCC	F20 | F-Z5
R05 | R-W3	5’- tataCGTCTCaTGCTCTCCAGCGCTTGTTTGCCACC	---AGCA 3’<br>3’ TCGT---	C-terminal Backbone (BsmBI site)	
R10 | R-X3	5’- tataCGTCTCaTGCTCTCAAGGGCTTGCTTGCCGC			
R15 | R-Y3	5’- tataCGTCTCaTGCTTTCCAAAGCCTGTTTTCCGCC			
R20 | R-Z3	5’- tataCGTCTCaTGCTTTCCAGTGCCTGCTTACCTCC			

1. The short and full names of each primer are separated by the symbol |.
2. The reverse and forward primers at the same line are compatible primer pairs whose PCR products could be ligated after USER™ or BsmBI digestion, while the primers at different lines are incompatible for ligation.
