# Supplementary material for: ULtiMATE System for Rapid Assembly of Customized TAL Effectors
Source: PLoS One. 2013 Sep 27;8(9):e75649. doi: 10.1371/journal.pone.0075649 (PMC3815405; doi:10.1371/journal.pone.0075649)
Supplement: Table S3 — gene targeting. (PDF) [file pone.0075649.s008.pdf]

## Supporting information, Table S3

**Table S3** Primers for colony PCR of TALE constructs and genome PCR verification of TALENs-mediated gene targeting

| Name of Primers | Sequence | Description |
|---|---|---|
| TAL-seq-F | 5’- AAGAGGGGAGGCGTGACGGC -3’ | For both colony PCR and sequencing analysis of the TALE constructs |
| TAL-seq-R | 5’- CAAGCCAGGGCCACCAGGT -3’ | |
| HBEGF-outer-F | 5’- TGAGTGAGCAAGACAAGACACTCAA -3’ | For nested genome PCR of cell clones harboring the pair of TALENs targeting *hHBEGF* |
| HBEGF-outer-R | 5’- GCATCTCCGCATGTGTTACCTCTATTTTATATATA -3’ | |
| HBEGF-inner-F | 5’- GTGGCCGCCGCTTCGAAAGTGAC -3’ | |
| HBEGF-inner-R | 5’- GTCCAAGGATGGGGGGCCTCCA -3’ | |
| ANTXR1-outer-F | 5’- TAAAAGTTGATGGTGATCCCACTTTCA -3’ | For nested genome PCR of cell clones harboring the pair of TALENs targeting *hANTXR1* |
| ANTXR1-outer-R | 5’- CTCCCTTCCTGGGTAATTATGAACAGTA -3’ | |
| ANTXR1-inner-F | 5’- CCAGCCTAGGAGGCCCCCTG -3’ | |
| ANTXR1-inner-R | 5’- GCACCAGAGCACAAATCTCAGGGCTA -3’ | |
| ANTXR2-outer-F | 5’- GACTCATATCATAGTTATGGTCATTAAGTCAAACT -3’ | For nested genome PCR of cell clones harboring the pair of TALENs targeting *hANTXR2* |
| ANTXR2-outer-R | 5’- ATCTAAACAAGAAAAATGTAGAACTACTTACAGTA -3’ | |
| ANTXR2-inner-F | 5’- TTATGGGTCATGGAGGAGTATGTGAGTGCT -3’ | |
| ANTXR2-inner-R | 5’- GCCTGAATCACCACTTGGAAATATCAATCATGC -3’ | |
| LRP1-outer-F | 5’- TAATACCATCTCCAACTACTACTGTGTGTG -3’ | For nested genome PCR of cell clones harboring the pair of TALENs targeting *hLRP1* |
| LRP1-outer-R | 5’- GTTAAAACAGCTGCCTCCGATTACA -3’ | |
| LRP1-inner-F | 5’- TGCACCCCCCTTCACCATCACTC -3’ | |
| LRP1-inner-R | 5’- GGAAGAGGGATAAGTTAAGGGTTTGGGGAGA -3’ | |
| ATG5-F | 5'- TAGCAGGTTCATTCATCGTTGCAAGGA -3’ | For genome PCR of cell clones harboring the pair of TALENs targeting *hATG5* |
| ATG5-R | 5'- ATGTAAGGAAAACAAAGTCCAGAACGC -3’ | |
| HSP90AB1-F | 5'- CAGATGATGCTGAGGCTGCTGGTC -3’ | For genome PCR of cell clones harboring the pair of TALENs targeting *hHSP90AB1* |
| HSP90AB1-R | 5'- GAGCCAACACGCTCAGCCACTTT -3’ | |
| PLXNA2-F | 5'- CTGGCCTGTGGGAGCCTCTA -3’ | For genome PCR of cell clones harboring the pair of TALENs targeting *hPLXNA2* |
| PLXNA2-R | 5'- CGGGTGGTGATACTGCTTCTGC -3’ | |
| VPS15-F | 5'- CTTGAATAACATTGAGAAGCGCTGGAT -3’ | For genome PCR of cell clones harboring the pair of TALENs targeting *hVPS15* |
| VPS15-R | 5'- ATAAACTGCTGTTTGCATTTCCTAGGC -3’ | |
| VPS34-F | 5'- TTGTTTTTCCTGTACCTAAGTTCCCGC -3’ | For genome PCR of cell clones harboring the pair of TALENs targeting *hVPS34* |
| VPS34-R | 5'- GCATCTGCCTGTGTCTACCTCTCCATA -3’ | |
