# Supplementary material for: ULtiMATE System for Rapid Assembly of Customized TAL Effectors
Source: PLoS One. 2013 Sep 27;8(9):e75649. doi: 10.1371/journal.pone.0075649 (PMC3815405; doi:10.1371/journal.pone.0075649)
Supplement: Text S1 — (DOCX) [file pone.0075649.s009.docx]

**Supporting Information: Text S1**

Kim HJ, Lee HJ, Kim H, Cho SW, Kim JS (2009) Targeted genome editing in human cells with zinc finger nucleases constructed via modular assembly. Genome Res 19: 1279-1288.
